# Comparative Transcriptomic Analysis Reveals Divergent Stress Adaptation Strategies in Gamma-Induced Soybean Mutants

**DOI:** 10.3390/plants15081241

**Published:** 2026-04-17

**Authors:** Tamer Gümüş, Sinan Meriç, Alp Ayan, Buse Özden, Simge Emlik, Özge Çelik, Alper Yılmaz, Çimen Atak

**Affiliations:** 1 Faculty of Science and Letters, Molecular Biology and Genetics, Istanbul Kültür University, Istanbul 34158, Türkiye; a.ayan@iku.edu.tr (A.A.); buse.ozden@iku.edu.tr (B.Ö.); s.emlik@iku.edu.tr (S.E.); ocelik@iku.edu.tr (Ö.Ç.); c.atak@iku.edu.tr (Ç.A.); 2 Graduate School of Science and Engineering, Bioengineering, Yıldız Technical University, Istanbul 34210, Türkiye; 3 Faculty of Arts and Sciences, Molecular Biology and Genetics, Yıldız Technical University, Istanbul 34220, Türkiye; alyilmaz@yildiz.edu.tr

**Keywords:** soybean, RNA-seq, isoflavonoid, salinity, drought, mutation breeding, SNP

## Abstract

Soybean (*Glycine max* L.) is an important agricultural crop for human food and animal feed. Soybean yield is severely constrained by abiotic stresses such as salinity and drought, which affect large proportions of arable and irrigated lands worldwide. This necessitates the development of new soybean varieties tolerant to these stress factors. Mutation breeding is an effective approach to improve the stress tolerance of plants due to increased genetic diversity. In this study, two gamma-induced salinity and drought-tolerant soybean mutants (SM1 and SM3-1) were compared with the parental line S04-05 using GO and KEGG pathway enrichment analyses. GO enrichment analyses revealed extensive differential gene expression in the mutant lines under stress conditions, with significant enrichment of pathways related to photosynthesis, hormone signaling, carbohydrate metabolism, and flavonoid and isoflavonoid biosynthesis. Genotype-specific analyses indicated that the SM3-1 mutant exhibited a dynamic regulatory response associated with maintaining the photosynthetic apparatus and chloroplast homeostasis under stress, whereas the SM1 mutant showed an adaptation strategy based on metabolite-mediated osmotic adjustment and ROS scavenging. Compared to the parental variety S04-05, the mutants showed distinct metabolic regulation in phenylpropanoid/isoflavone metabolism, with upregulation of many isoflavone biosynthesis genes under salinity, drought, and untreated conditions, indicating a key and sustained role of this pathway in stress tolerance. Most SNPs identified in the isoflavone biosynthesis pathway consist of moderate-impact and modifier variations. These findings suggest that gamma mutagenesis and subsequent selection processes allow for the development of novel genetic variants that operate through different physiological and metabolic mechanisms but exhibit similar levels of tolerance. In this respect, the study reveals that mutation breeding is a potentially sustainable and effective breeding strategy for increasing abiotic stress tolerance in soybeans.

## 1. Introduction

Soybeans (*Glycine max* (L.) Merrill), native to northern China, are an annual plant belonging to the legume family and are one of the world’s most important crops due to their exceptional agricultural and industrial properties. Agriculturally, soybeans are prized for their roughly 40% protein (all essential amino acids), 20% fats (largely unsaturated), 30% carbohydrates, 17% fibers (both soluble and insoluble), and 5% ash as well as minerals such as calcium, magnesium, potassium, iron, and zinc which makes them a key source of plant-based nutrition for both humans and livestock [1]. Soybeans are used in the production of a wide variety of food products, including vegetable oil, biodiesel, animal feed, tofu, soy milk, and textured plant protein, as well as in the manufacture of inks, plastics, adhesives, and cosmetics, highlighting their role as a renewable resource supporting both the agricultural economy and numerous industrial sectors [2].

Over the past 100 years, since 1900, the global climate has warmed by approximately 0.8 °C due to greenhouse gas emissions and is estimated to increase by 2–5 °C by the end of the 21st century [3]. It is anticipated that such drastic changes will worsen drought conditions and water scarcity, with consequent effects on agriculture, forests and vegetation, clean water resources, sea levels, energy, human health, and biodiversity [4]. The term “arid” is generally used to describe areas that experience drought during periods of low rainfall [5]. Drought can be broadly classified into meteorological, agricultural, and hydrological drought [6]. Intense sunlight and wind are associated with meteorological drought, which can result in severe dehydration, wilting, reduced growth, and even plant death, especially in young herbaceous plants [7]. While hydrological drought occurs when there is insufficient water resources, agricultural drought results from insufficient soil moisture to meet the water needs of plants [6]. In addition to drought, salinity is another major factor limiting soil fertility, particularly in arid regions, where limited rainfall and drought-induced evaporation led to salt accumulation in the soil [8,9]. Approximately 33% of the world’s agricultural land is known to be saline, and this percentage is increasing each year [10]. Since most economically valuable plants are sensitive to soil salinity, it has become imperative to develop plants that are high in nutritional value and have a wide range of tolerance not only to salt but also to all other types of abiotic stresses [11,12].

In eukaryotic organisms, the main factor causing genetic variation is crossing-over during meiosis, which is influenced by the available gene pools and hybridization within populations. However, the gene pool of soybeans, which undergoes closed fertilization, is smaller than that of species that reproduce through open fertilization. Therefore, more extensive breeding efforts are required to bring about a desired trait. Inducing genetic variations in the genome is a prerequisite step in breeding programs for closed-fertilizing plants like soybeans [13]. Today, methods such as selection breeding, hybrid breeding, mutation breeding, and biotechnological breeding are used in the development of cultivated plants. The most important factor in plant breeding is the induction of genetic variation. Today, plant breeders use several techniques in combination to increase the effectiveness of breeding and shorten the necessary long periods. One of the easiest ways to create genetic variation is to induce mutations [14]. Mutations represent heritable changes in the DNA sequence that are not caused by genetic recombination, resulting from changes in the physical and chemical structure of genetic material. In the 60 years since the pioneering works carried out in the 1960s, 3222 plant mutant varieties from 170 different plant species in more than 60 countries throughout the world have been obtained through direct mutation and the hybridization of mutants [15]. In mutation breeding, the primary strategy is to develop new varieties with improved traits by modifying one or more traits of an established variety without altering its established characteristics. When comparing, mutation breeding results in very few changes in the genotype of the variety compared to hybrid breeding. The long time required in classical hybrid breeding studies is partially eliminated in mutation breeding [14].

Flavonoids are the largest group of naturally produced substances, with over 9000 phenolic compounds identified in plants [16]. Important flavonoid subgroups include anthocyanins, chalcones, flavonols, flavanols, flavanonols, flavanones, flavones, and isoflavonoids [17]. Flavonoids are key secondary metabolites that enhance the resistance of plants to both biotic and abiotic stress. Plants under stress accumulate flavonoids through the regulation of flavonoid synthase genes’ expression [18]. Several key candidate genes and metabolites involved in isoflavone biosynthesis play significant roles in the response of soybeans to both polyethylene glycol (PEG)-simulated drought stress (osmotic stress) [19] and water deficit [20]. Some aspects of drought resistance in soybean varieties are differences in the content of major metabolic pathways and metabolites, including flavonoids, within these metabolic pathways. Similarly, under salt stress, salt-tolerant plants have higher levels of most flavonoid metabolites compared to salt-sensitive plants, and the total flavonoid content of salt-tolerant plants is significantly higher than that of salt-sensitive plants [21]. Additionally, a greater number of differentially expressed genes (DEGs) were detected in salt-tolerant soybeans under salt stress compared to salt-sensitive soybeans, and KEGG (Kyoto Encyclopedia of Genes and Genomes) enrichment analysis showed that DEGs were primarily enriched in pathways related to phenylpropanoid biosynthesis, isoflavonoid biosynthesis, flavonoid biosynthesis, and also flavone and flavonol biosynthesis [21]. Varieties with high flavonoid accumulation tend to be more tolerant to salt than those with low accumulation [22]. The synthesis of major flavonoid classes, including anthocyanins, isoflavonoids, and proanthocyanidins, occurs along the general phenylpropanoid and polyketide pathways [23].

In summary, abiotic stresses such as salinity and drought severely limit soybean yield, and the development of genotypes tolerant to these stresses is one of the main goals of current plant breeding studies. Mutant lines developed by gamma irradiation offer an important model for studying the molecular basis of stress tolerance due to their increased genetic diversity. However, it is not yet fully understood which regulatory and metabolic networks govern salinity and drought tolerance in soybean mutants developed through mutation breeding, including in vivo and in vitro selection, and which molecular strategies enable different mutant genotypes to achieve similar levels of stress tolerance. In this study, the gamma-induced SM1 and SM3-1 mutant lines, identified as tolerant to salinity and drought, were compared with the parental line S04-05. The main objectives of this study were (i) to characterize genotype-specific transcriptome responses under stress conditions, (ii) to investigate the regulation of stress-related metabolic pathways, particularly isoflavonoid biosynthesis, and (iii) to explore potential associations between SNP (single nucleotide polymorphism) variation, gene expression, and stress adaptation. It is expected that these findings will contribute to understanding the molecular basis of stress tolerance in soybean lines developed through mutation breeding and provide valuable genetic resources for future breeding efforts.

## 2. Results

### 2.1. Identification of Differentially Expressed Genes Using RNA Sequencing

**Figure 1:**
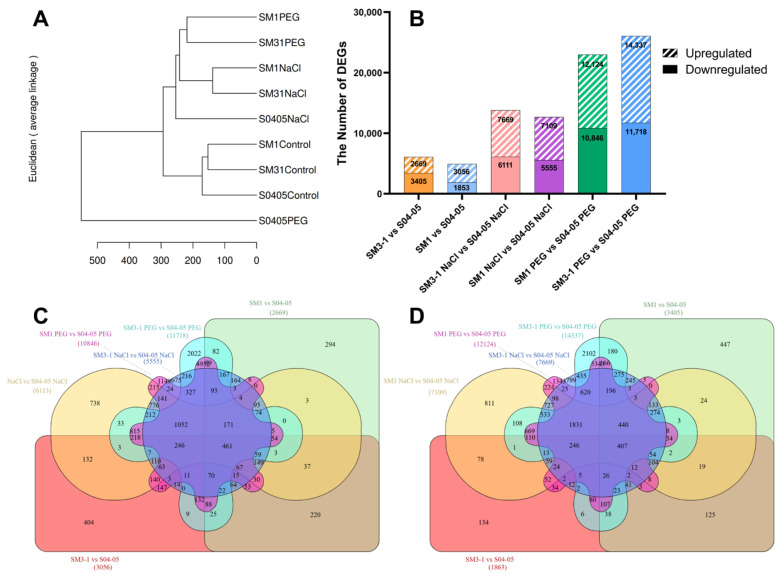
(**A**) Hierarchical clustering analysis based on gene expression profiles. (**B**) Number of differentially expressed genes (DEGs) in pairwise comparisons of the original variety and mutants under salt and drought treatments. Bars show total DEGs (upregulated + downregulated) for each comparison with |log2FC| ≥ 1 and FDR < 0.05. (**C**) Venn diagram presenting genes whose expression is increased under optimal conditions and treatment in the original variety and mutant lines. Overlapping regions show shared upregulated genes among comparisons. Numbers in each segment indicate unique and shared gene counts. (**D**) Venn diagram presenting genes whose expression is decreased under optimal conditions and treatment in the original variety and mutant lines. Overlapping regions show shared downregulated genes among comparisons. Numbers in each segment indicate unique and shared gene counts. The central overlap regions in panels (**C**,**D**) represent genes constitutively up- or downregulated in both S04-05 and mutant lines (SM3-1 and SM1) across all conditions. The numbers of genes at all intersection sets in both Venn diagrams are given in Supplementary S1.

In the hierarchical clustering analysis based on the gene expression profiles of the samples, two main branches were observed. All stressed mutant samples (NaCl and PEG) clustered in the first main branch. The NaCl treatments of the mutant lines (SM1 NaCl and SM3-1 NaCl) presented close clustering among themselves. Also, the PEG treatments of the mutants (SM1 PEG and SM3-1 PEG) formed a sub-cluster close to these. S04-05 NaCl, however, was found in a separate branch from this experimental group. The second main branch contained all untreated groups (S04-05, SM1 and SM3-1) and was distinctly different from the stress groups. Finally, S04-05 PEG was positioned separately from these two main branches, revealing the most distinct expression profile (Figure 1A).

Statistical graphs presenting the number of DEGs in the dual comparison of original varieties and mutants under NaCl and PEG treatments are provided. Both mutant samples presented an increased number of upregulated and downregulated genes in response to PEG treatment. In SM3-1 mutants, 2669 genes were upregulated, and 3405 genes were downregulated compared to S04-05. Similarly, in SM1, 3056 genes were upregulated, and 1853 genes were downregulated compared to S04-05. In SM3-1 NaCl samples, 7699 genes were upregulated, and 6111 genes were downregulated compared with S04-05 NaCl. Compared with S04-05 NaCl, SM1 NaCl had 7109 upregulated and 5555 downregulated genes. Among PEG treatments, SM1 PEG presented 12,124 upregulated and 10,846 downregulated genes compared to S04-05 PEG. Between the SM3-1 PEG and S04-05 PEG groups, 14,337 genes were upregulated and 11,718 genes were downregulated. Interestingly, in all comparisons, the number of upregulated genes was found to be higher than the number of downregulated genes, except for in the SM3-1 vs. S04-05 comparison (Figure 1B). Venn diagrams of the increased and decreased gene expressions for S04-05 and mutants under salt and drought treatments are shown in Figure 1C,D. In all comparisons, the number of commonly increased genes was 461, while the number of commonly decreased genes was 407.

**Figure 2:**
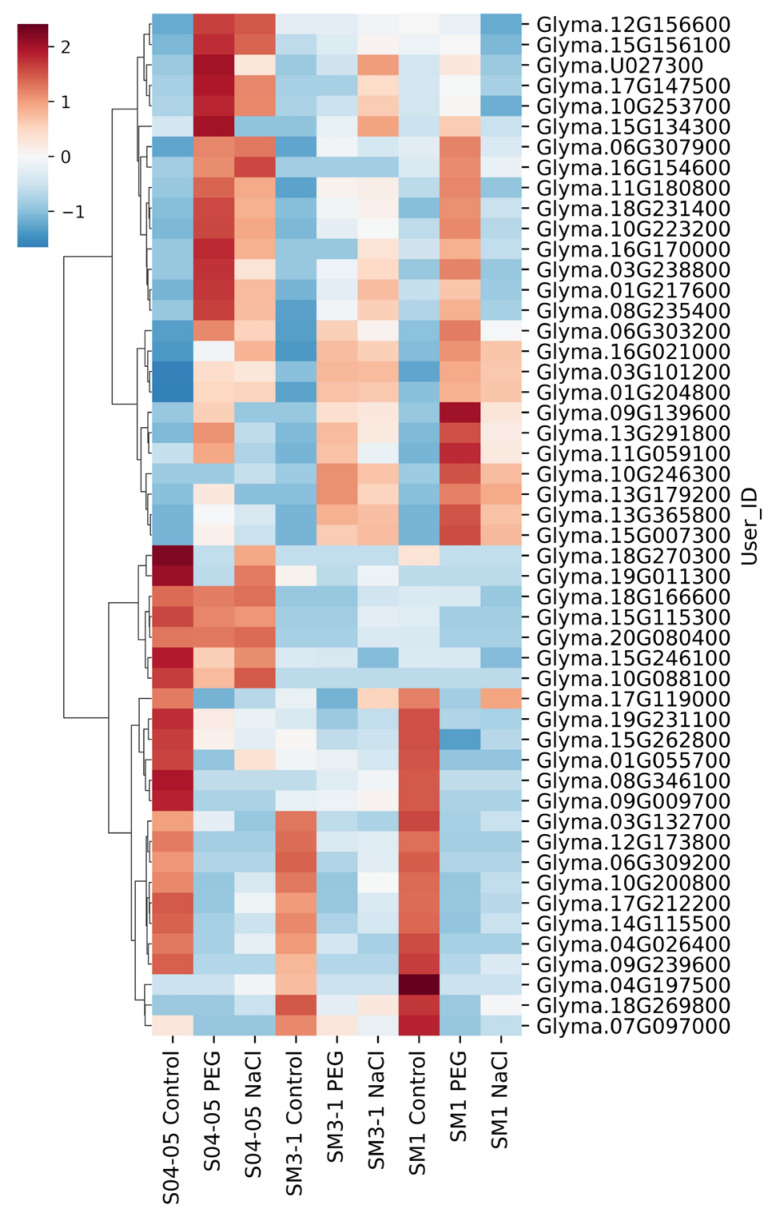
Expression profiles of the 50 genes with the highest row-wise variance (on log_2_-transformed counts) in the S04-05 variant and the SM1 and SM3-1 mutant lines under optimal conditions and NaCl and PEG treatments. Heatmap based on z-score normalized expression values.

Expression profiles of the 50 most variable genes under different stress conditions and genotypes are shown using RNA-seq data as row-wise normalized z-score values (Figure 2). The heatmap results revealed that the DEGs with the highest variance showed significant differences between mutants and the original variety. Both SM1 and SM3-1 mutants exhibited altered gene expression profiles compared to the parental variety under both stress conditions. In the z-score-based heatmap, untreated samples formed a distinct cluster, separated from PEG and NaCl treatments. Although SM1 and SM3-1 genotypes exhibited partially similar expression profiles under stress conditions, genotype-specific expression differences were noted in some genes.

### 2.2. Functional Annotation of Identified DEGs Induced by Salt and Drought Stress

**Figure 3:**
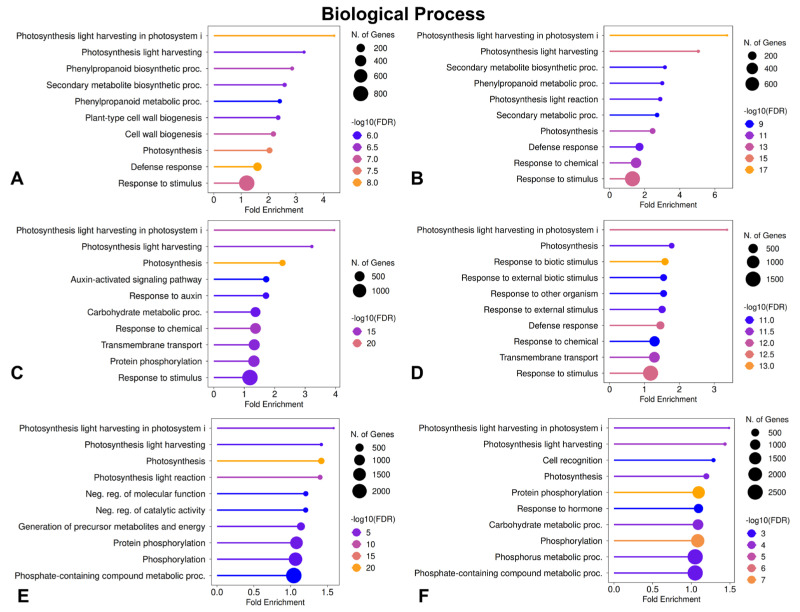
Biological Process (BP) Gene Ontology enrichment analysis of genotype and stress condition-related differentially expressed genes. Cellular Component (CC) and Molecular Function (MF) enrichment results are provided in Supplementary S2. (**A**) Untreated SM3-1 vs. untreated S04-05, (**B**) untreated SM1 vs. untreated S04-05, (**C**) SM3-1 NaCl vs. S04-05 NaCl, (**D**) SM1 NaCl vs. S04-05 NaCl, (**E**) SM3-1 PEG vs. S04-05 PEG, (**F**) SM1 PEG vs. S04-05 PEG. Significantly enriched pathways (FDR < 0.05) are visualized using a lollipop plot. In this plot, each pathway is represented by a line and a dot, where the length of the line reflects the enrichment level, the dot size indicates the number of genes in that pathway, and the color scale indicates statistical significance −log10(FDR).

To reveal transcriptomic differences between different genotypes and stress conditions, GO enrichment analyses were performed for DEGs. The analyses were evaluated under the categories of biological processes (BPs) (Figure 3), cellular components (CCs), and molecular functions (MFs). Figures for the CC and MF categories are given in Supplementary S2. In the comparison made under optimal growth conditions (absence of stress), the results showed that photosynthesis, secondary metabolite biosynthetic processes, light harvesting, and chloroplast-related BPs were significantly enriched in the SM3-1 mutant line (Figure 3A). In the SM1 mutant, under optimal growth conditions, BPs related to photosynthesis, secondary metabolic processes, and response to stimulus were enriched (Figure 3B).

Under NaCl treatment, the SM3-1 mutants showed a substantial enrichment of BPs related to photosynthesis, as well as response to stimulus, hormone responses, and transmembrane transport processes, compared with the S04-05 lineage (Figure 3C). Under NaCl treatment, the SM1 mutants revealed an enrichment of BPs related to defense response, chemical stimuli response, and ion transport compared to the S04-05 lineage (Figure 3D). Under PEG conditions, the SM3-1 mutants indicated a substantial enrichment of BPs related to photosynthesis, energy metabolism, and phosphorylation compared to the S04-05 line (Figure 3E). Under PEG treatment, the SM1 mutants presented enrichment of BPs related to hormone response and carbohydrate metabolism compared with S04-05 (Figure 3F).

**Figure 4:**
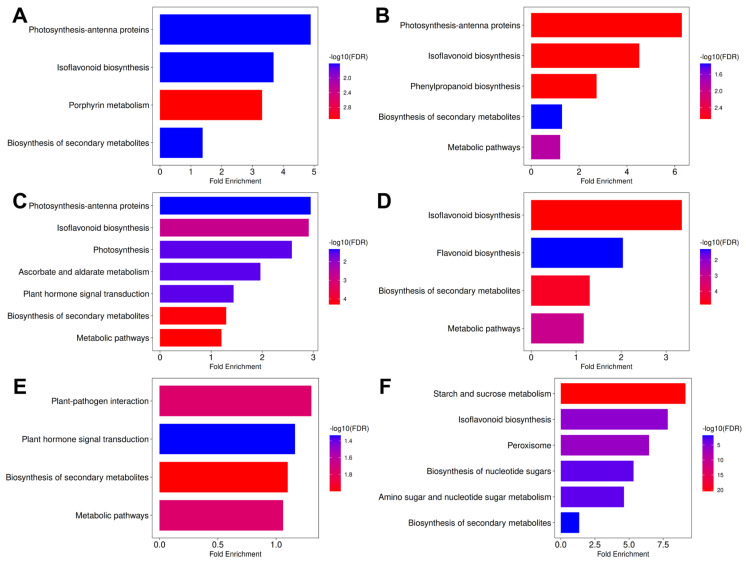
KEGG pathway enrichment analysis of genotype and stress condition-related differentially expressed genes. (**A**) Untreated SM3-1 vs. untreated S04-05, (**B**) untreated SM1 vs. untreated S04-05, (**C**) SM3-1 NaCl vs. S04-05 NaCl, (**D**) SM1 NaCl vs. S04-05 NaCl, (**E**) SM3-1 PEG vs. S04-05 PEG, (**F**) SM1 PEG vs. S04-05 PEG. Bar plots display significantly enriched pathways (FDR < 0.05) with bar size indicating fold enrichment and color representing −log10(FDR).

Considering all results, GO enrichment analyses show that the mutant lines are significantly different from S04-05 under both untreated and stress conditions. Although SM1 and SM3-1 activate different mechanisms depending on the condition, they exhibit a similar overall profile. Compared to S04-05 under optimal growth conditions, the most significant enrichments in both mutant lines were in photosynthesis-antenna proteins, phenylpropanoid biosynthesis, and isoflavonoid biosynthesis pathways (Figure 4A,B). Under NaCl treatment, secondary metabolite and isoflavonoid biosynthesis were strongly stimulated in both mutants. At this point, a divergence between the two lines is noticeable: photosynthesis and hormone signaling pathways are prominent in SM3-1, while flavonoid biosynthesis stands out as a decisive difference in SM1 (Figure 4C,D). In the comparison of the S04-05 versus SM3-1 and SM1 mutants under PEG treatment, secondary metabolite biosynthesis was common as well. While hormone signaling pathways were enriched in SM3-1, starch–sucrose metabolism, amino sugar/nucleotide sugar metabolism, and isoflavonoid biosynthesis showed significant enrichment in SM1 (Figure 4E,F). All these findings indicate that phenylpropanoid/isoflavone metabolism is significantly reorganized in the mutant lines. Therefore, a KEGG enrichment analysis was performed to provide a comparative evaluation of the isoflavonoid biosynthesis pathway and the key genes involved in this pathway in order to illuminate their possible roles in stress tolerance.

**Figure 5:**
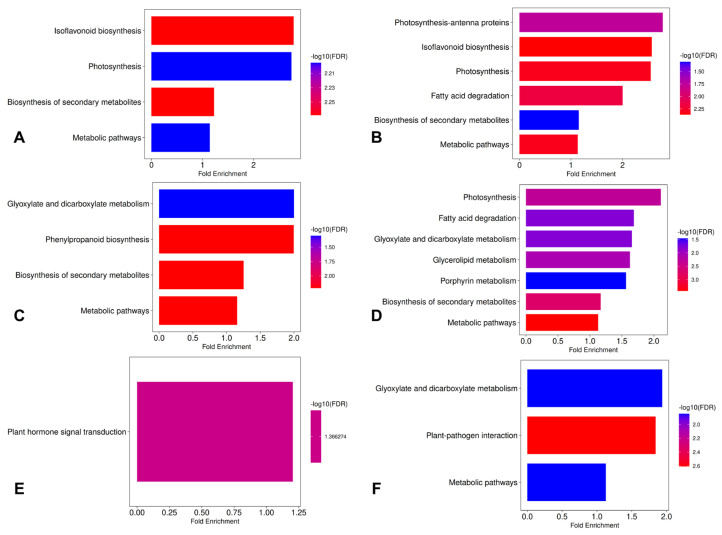
KEGG pathway enrichment analysis of genotype and stress condition-related differentially expressed genes for mutant lines. (**A**) SM3-1 NaCl vs. untreated SM3-1, (**B**) SM1 NaCl vs. untreated SM1, (**C**) SM3-1 PEG vs. untreated SM3-1, (**D**) SM1 PEG vs. untreated SM1, (**E**) SM3-1 PEG vs. SM3-1 NaCl, (**F**) SM1 PEG vs. SM1 NaCl. Bar plots display significantly enriched pathways (FDR < 0.05) with bar size indicating fold enrichment and color representing −log10(FDR).

KEGG enrichment analysis was performed to determine the role of mutants in stress responses in detail. Under NaCl treatment, the strongest DEG enrichment was observed in metabolic pathways, photosynthesis, and isoflavonoid biosynthesis pathways in both mutant lines compared to optimal growth conditions, paralleling the comparative patterns observed for the mutant lines and S04-05 in Figure 4 (Figure 5A,B). In addition, the fatty acid degradation pathway was prominent in the SM1 mutant. Under PEG treatment, both SM3-1 and SM1 mutants showed enrichment in glyoxylate and dicarboxylate metabolism, metabolic pathways, and the biosynthesis of secondary metabolites. Furthermore, phenylpropanoid biosynthesis was prominent in the SM3-1 mutant, while extensive activation was observed in photosynthesis, lipid metabolism, and porphyrin metabolism pathways in the SM1 mutant (Figure 5C,D). In a direct comparison between the two stress conditions, PEG and NaCl, only the plant hormone signaling pathway was enriched in the SM3-1 mutant, while glyoxylate metabolism and plant–pathogen interaction and metabolic pathways were enriched in the SM1 mutant (Figure 5E,F).

**Figure 6:**
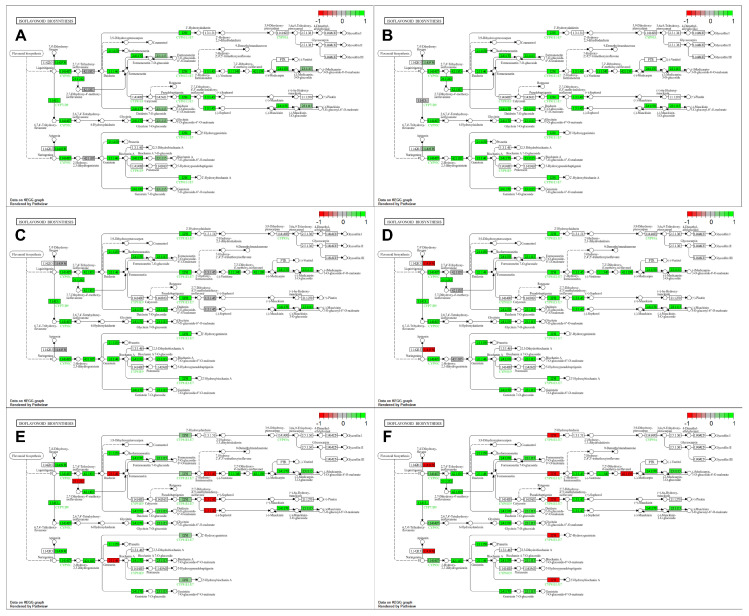
Isoflavonoid biosynthesis pathway comparison of genotype and stress condition-related gene expression. Expression levels of key pathway genes are shown for parent S04-05 and mutant lines SM1 and SM3-1 under untreated, NaCl treatment, and PEG treatment conditions. Gene expression changes were visualized as colored boxes placed over each enzymatic step. (**A**) Untreated SM3-1 vs. untreated S04-05, (**B**) untreated SM1 vs. untreated S04-05, (**C**) SM3-1 NaCl vs. S04-05 NaCl, (**D**) SM1 NaCl vs. S04-05 NaCl, (**E**) SM3-1 PEG vs. S04-05 PEG, (**F**) SM1 PEG vs. S04-05 PEG. The images in Figure 6 are presented separately in a larger size in Supplementary S3.

KEGG pathway analyses of DEGs show expression changes in various enzymatic steps involving 32 DEGs in the isoflavonoid biosynthesis pathway, depending on the genotype and stress conditions. Under optimal growth conditions, SM3-1 and SM1 mutants showed an increased expression trend across the entire isoflavonoid biosynthesis pathway compared to S04-05 (Figure 6A,B). Under NaCl treatment, compared to S04-05, the expression of the cytochrome P450 ([EC:1.14.19.76], Glyma.12G067100) and 2′-hydroxyisoflavone reductase ([EC:1.3.1.45], Glyma.01G211800) gene in the isoflavonoid biosynthesis pathway remained unchanged in SM3-1. In SM1, cytochrome P450 ([1.14.19.76]) showed decreased expression. 2-hydroxyisoflavanone dehydratase ([EC:4.2.1.105], Glyma.10G250300) showed unchanged expression levels, while other genes are increased in the pathway (Figure 6C,D). Under PEG treatment, compared to S04-05, the expression of genes in the enzymatic steps of isoflavone 4′-O-methyltransferase ([EC:2.1.1.212 2.1.1.46], Glyma.13G173600 and Glyma.13G173300), 2′-hydroxyisoflavone reductase ([EC:1.3.1.45], Glyma.01G211800 and Glyma.11G070500) decreased in SM3-1, while the expression of 28 genes in the other enzymatic steps increased. In the SM1 mutant line under PEG treatment, the expression of genes in the enzymatic steps cytochrome P450 ([EC:1.14.19.76], Glyma.12G067000), isoflavone 2′-hydroxylase ([EC:1.14.14.90 1.14.14.89], Glyma.09G048700) and pterocarpan synthase ([EC:4.2.1.139] Glyma.19G151100) was decreased, while the expression of 26 other genes was increased (Figure 6E,F).

**Figure 7:**
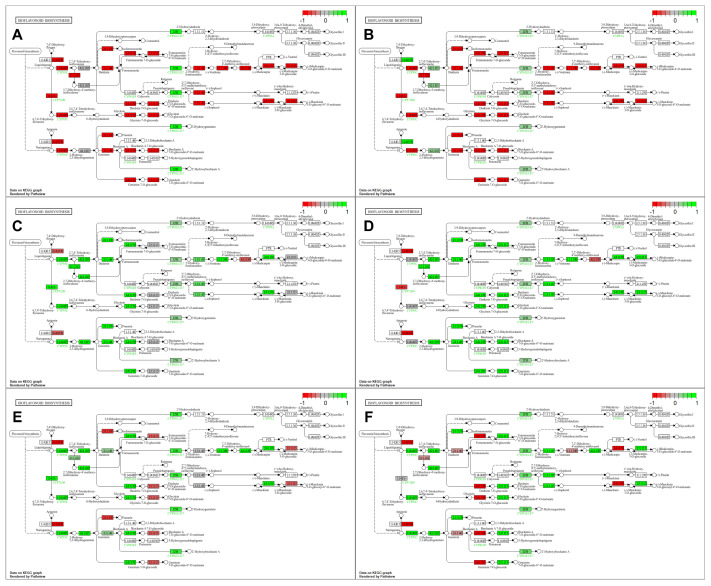
Isoflavonoid biosynthesis pathway comparison of genotype and stress condition-related gene expression. Expression levels of key pathway genes are shown for parent S04-05 and mutant lines SM1 and SM3-1 under untreated, NaCl treatment, and PEG treatment conditions. Gene expression changes were visualized as colored boxes placed over each enzymatic step. (**A**) S04-05 NaCl vs. untreated S04-05, (**B**) S04-05 PEG vs. untreated S04-05, (**C**) SM3-1 NaCl vs. untreated SM3-1, (**D**) SM1 NaCl vs. untreated SM1, (**E**) SM3-1 PEG vs. untreated SM3-1, (**F**) SM1 PEG vs. untreated SM1. The images in Figure 7 are presented separately in a larger size in Supplementary S3.

In the original variety S04-05, under NaCl treatment, the expression of the enzymatic step 2-hydroxyisoflavanone dehydratase ([EC:4.2.1.105], Glyma.10G250300) remained unchanged, the enzymatic step of isoflavone 2′-hydroxylase ([EC:1.14.14.90 1.14.14.89], Glyma.09G048700) increased, and all other genes expressed in the pathway showed decreased expression. In the original variety S04-05, under PEG treatment, all pathways except cytochrome P450 ([EC:1.14.19.76], Glyma.12G067000), 2-hydroxyisoflavanone dehydratase ([EC:4.2.1.105], Glyma.10G250300) and isoflavone 2′-hydroxylase ([EC:1.14.14.90 1.14.14.89], Glyma.09G048700) enzymatic steps showed a decreased expression profile (Figure 7A,B). In SM3-1 mutants, under salinity stress, an increased profile was observed at the whole pathway level compared to the untreated SM3-1 mutants, while cytochrome P450 ([EC:1.14.19.76], Glyma.12G067000 and Glyma.12G067100) and pterocarpan synthase ([EC:4.2.1.139], Glyma.03G147700) enzymatic steps showed decreased expression, and there was no change in the isoflavone 7-O-glucoside-6″-O-malonyltransferase ([EC:2.3.1.115], Glyma.19G030500 and Glyma.19G030500) enzymatic step. SM1 salinity treatment showed decreased expression in three enzymatic steps of cytochrome P450 ([EC:1.14.19.76], Glyma.12G067000), isoflavone-7-O-methyltransferase ([EC:2.1.1.150], Glyma.09G09460 and Glyma.08G248000), and isoflavone 7-O-glucoside-6″-O-malonyltransferase ([EC:2.3.1.115], Glyma.19G030500) compared to untreated SM1 samples, while the 2′-hydroxyisoflavone reductase ([EC:1.3.1.45], Glyma.01G211800) step remained unchanged. (Figure 7C,D). In SM3-1, under PEG treatment, cytochrome P450 ([EC:1.14.19.76], Glyma.12G067000 and Glyma.12G067100) and pterocarpan synthase ([EC:4.2.1.139], Glyma.03G147700) enzymatic steps showed decreased expression, while the 2-hydroxyisoflavanone synthase ([EC:1.14.14.87], Glyma.13G173500) enzymatic step remained unchanged in SM1 mutants. Under PEG treatment, the enzymatic step of cytochrome P450 ([EC:1.14.19.76], Glyma.12G067000), isoflavone 4′-O-methyltransferase ([EC:2.1.1.212 2.1.1.46], Glyma.13G173300), isoflavone 7-O-glucosyltransferase ([EC:2.4.1.170], Glyma.16G175600 and Glyma.16G175900), and vestitone reductase ([EC:1.1.1.348], Glyma.18G220500) showed decreased expression, while flavonoid 6-hydroxylase ([EC:1.14.13.-] Glyma.18G080400 and Glyma.11G108300) remained the same (Figure 7E,F). Under PEG treatment, different pathway-level regulatory patterns emerged in the SM3-1 and SM1 mutants. More extensive changes were observed in both mutant lines compared to NaCl treatment, and it was determined that pathway flow was reshaped by upregulation or downregulation of different enzyme steps. Overall, KEGG analyses revealed that PEG treatment has a stronger pathway-level effect in mutant lines compared to NaCl treatment.

### 2.3. SNP Analysis

**Figure 8:**
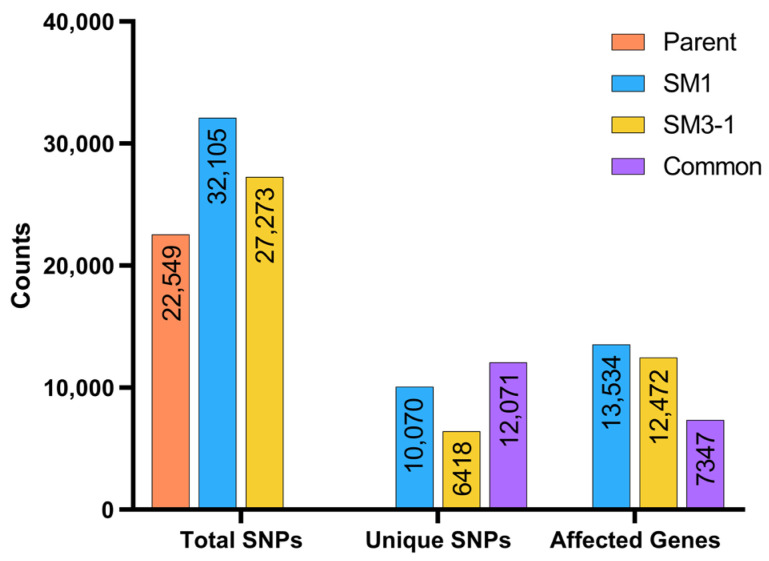
Distribution of single-nucleotide polymorphisms and affected genes in genotype-based comparisons. Total SNP counts, unique SNP counts, and number of affected genes are shown for parent cultivar S04-05, mutant lines SM1 and SM3-1, and shared variants between SM1 and SM3-1 genotypes.

When the SNP results were examined, a total of 22,549 SNPs were found in the S04-05 variety, 27,273 in the SM3-1 mutant and 32,105 in the SM1 mutant according to the reference genome (Glycine max Wm82.a2.v1). Comparative SNP analysis revealed 10,070 unique SNPs in SM1 and 6418 unique SNPs in SM3-1, with both mutants sharing 12,071 SNPs compared to the S04-05 variety. These SNPs were distributed across 13,534 genes in the SM1 mutant, 12,472 genes in the SM3-1 mutants, and 7347 genes in both mutants (Figure 8).

**Figure 9:**
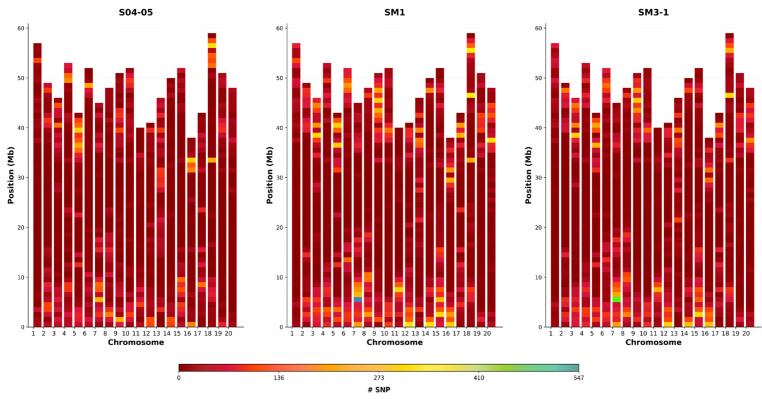
Chromosomal distribution of single SNPs in genotype-based comparisons. SNP positions are shown across all 20 chromosomes for the parental cultivar S04-05 and mutant lines SM1 and SM3-1. Color intensity represents SNP density per genomic window (e.g., 1 Mb bins) for each genotype. Genomic regions are color-coded based on SNP density using a continuous gradient: warmer colors (dark red) indicate low SNP density, intermediate colors (orange to yellow) represent moderate SNP density, and cooler colors (green to blue) represent high SNP density (hotspots).

A graph presenting chromosome lengths (Mb) was drawn, containing a 1 Mb window size, to determine the distribution of SNPs on chromosomes. The examination of the graph revealed that SNPs were particularly concentrated at the ends of chromosomes. Chromosomes 7, 15 and 18 were identified as containing the highest number of SNPs (Figure 9).

SNP analyses have revealed multiple variations in numerous genes within the isoflavone biosynthesis pathway. While some of these SNPs in the genes involved in the isoflavone biosynthesis pathway were observed in both mutant lines, others were determined to be specific to one of the mutants (Table 1). In the MYB transcription factor gene family (GmMYB176), various SNPs were detected in both mutant lines. These variants included a 3′UTR variant and two different missense mutations (G232D and N46T). Moreover, another MYB transcription factor (CCA1-like) gene contained a missense (A21V) mutation arising from a C/T SNP. Additionally, an upstream gene variant mutation occurred in the isoflavone reductase 4 (IFR4) gene in the SM1 mutant, resulting from a T/C SNP. In the isoflavone synthase 2 (IFS2) gene, a missense mutation (K340N) was detected in both SM1 and SM3-1 mutants. The flavonoid 6-hydroxylase (F6H) gene was identified as another missense mutation (T248A) that was seen in the only SM3-1 mutant. In the isoflavone 7-O-glucosyltransferase (UGT7) gene, a missense mutation (E428D) was identified in both mutant lines. Moreover, genes containing SNPs and their paralogs, which are provided in Supplementary S4, show a simultaneous expression pattern under all conditions.

### 2.4. Transcription Binding Site Analysis

To investigate the transcriptional regulation of isoflavone biosynthesis genes, a transcription factor binding site (TFBS) analysis was performed on the 15 nucleotides upstream and downstream of the SNP in genes containing SNPs. In the GmMYB176 gene (Glyma.05G032200), no TF binding motifs were identified in the S04-05, SM3-1, and SM1 lines. The SM3-1 mutant contains a T/C SNP in the upstream region of the IFR4 gene (Glyma.11G070600). Three TF binding motifs were identified in S04-05, SM3-1, and SM1, and these regions did not include the location of the SNP. TFBSs identified in the IFR4 gene include transcription factor family, PlantPAN similarity score, chromosomal position (start-end), strand orientation, and consensus binding motif sequence. TFmatrix ID information for each TFBS is provided in Supplementary S5.

### 2.5. Homology Modelling

**Figure 10:**
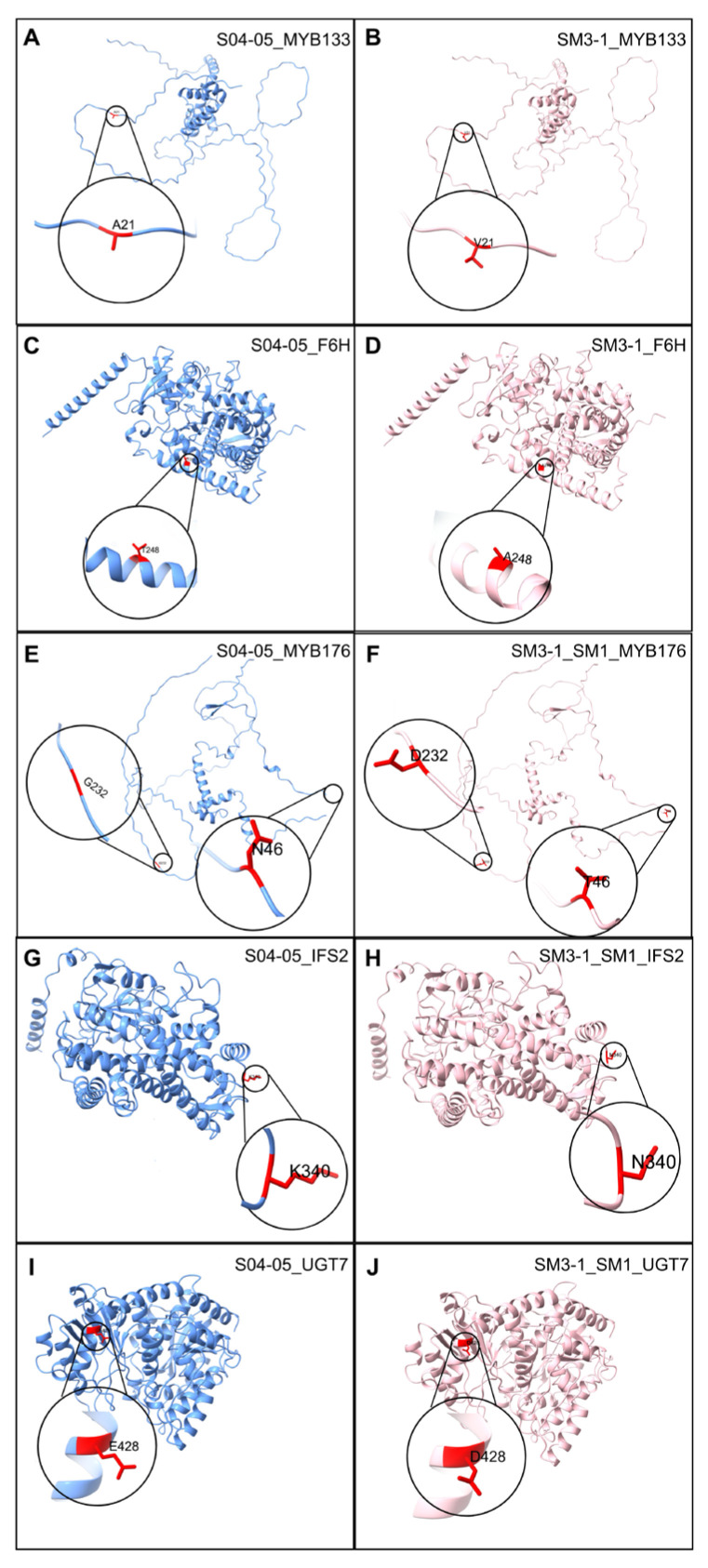
Predicted protein three-dimensional structural models of selected proteins in the isoflavonoid biosynthesis pathway showing SNP-induced amino acid changes. (**A**,**B**) MYB133: A21V mutation in SM3-1. (**C**,**D**) F6H: T248A mutation in SM3-1. (**E**,**F**) MYB176: G232A and N46T mutations in mutant lines (SM3-1/SM1). (**G**,**H**) IFS2: K340N mutation in mutant lines (SM3-1/SM1). (**I**,**J**) UGT7: E428D mutation in mutant lines (SM3-1/SM1). Parent structures (blue) and mutant structures (pink) are shown with mutation sites highlighted in red stick models. Mutated residues are shown in magnified insets.

Homologous models were constructed to determine whether structural changes in isoflavone biosynthesis pathway proteins were caused by SNP-derived variants and compared with the reference proteins. In all five genes, the mutant models closely resembled the three-dimensional structures of the corresponding reference proteins (Figure 10). Consequently, no significant conformational differences were observed. The selected templates and model quality criteria are given in the Supplementary S6.

### 2.6. Conservation Analysis

Multiple Sequence Alignment (MSA) was performed among homologs of *MYB176*, *MYB133*, *F6H, UGT7* and *IFS2* from 72, 73, 49 and 43 species, respectively, to determine the evolutionary conservation of SNP-containing residues in proteins involved in isoflavone biosynthesis pathways. Alignment-based conservation analysis showed that these SNP regions were subject to varying levels of restriction (Table 2). The highest conservation was observed in *UGT7* (dominant residue E, 95.24%), while the greatest variability was detected in *MYB133*/21 (dominant residue G, 38.98% with higher entropy and a greater number of variants). The remaining SNP positions (*MYB176*/46, *IFS2*/340 and *F6H*/248) showed conservation in the range of 43–90%. Additionally, amino acid distributions at positions containing MSAs and SNPs are visualized as bar graphs in Supplementary S6.

## 3. Discussion

Global climate change negatively impacts agricultural production, especially by increasing the duration and intensity of abiotic stresses such as drought and salinity. New breeding approaches are needed to increase plant resilience under these conditions, and mutation breeding stands out as a key tool in developing stress-tolerant genotypes. Transcriptome analyses of the present study showed that gamma-irradiated mutant lines exhibited significantly altered gene expression patterns compared to the parental S04-05 soybean variety, which is consistent with the literature reporting that physical mutagens induce random but intense genome-wide genetic changes, with broad transcriptomic, proteomic, and metabolomic implications [24,25]. In this study, the transcriptome profiles of the SM1 and SM3-1 mutants were clearly distinct from those of the parental S04-05 breeding line. This suggests that physical mutagens such as gamma-rays produce broad effects on gene regulatory regions and coding sequences [26,27]. Gamma- and heavy-ion mutagenesis are proven to generate a diverse spectrum of mutations on the whole genome by resequencing and transcriptomic studies [28,29]. Proteomic analyses of Meriç et al. (2023) [30] reported significantly altered protein profiles in gamma-induced mutants compared to parental lines, demonstrating that this differentiation arises from gamma-induced mutations in sequences associated with multiple biological processes. Therefore, the extensive transcriptomic differences observed in this present study are proposed to be due to regulatory network changes resulting from gamma-irradiation-induced DNA breaks, rearrangements, and point mutations and are the primary mechanism that broadens the stress response capacity of the mutant lines.

Based on the results, all samples subjected to NaCl and PEG treatments were clustered into a single main cluster, while the untreated groups were clustered into a separate major cluster (Figure 1A). This suggests that osmotic and oxidative stresses initiate early and strong molecular responses. Similar clustering structures have been reported in *Arabidopsis thaliana* [31], *Oryza sativa* [32], and *Glycine max* [20,33,34,35] under salinity and drought, confirming that stress regulates the transcriptome in a dominant manner. Our results indicated that both parental and mutant lines activated shared pathways under stress conditions. However, the activation was more extensive in the mutants. Therefore, the clustering results suggest that stress-related regulatory networks may be activated more rapidly and robustly in the lines developed through mutation breeding [36,37].

The fact that SM1 and SM3-1 mutant lines exhibited a higher number of DEGs compared to the parental lines under both NaCl and PEG treatments suggests that gamma mutagenesis expands the stress response capacity (Figure 1B). The more extensive gene expression changes observed in mutant lines are also consistent with studies reporting that genetic variation induced by gamma irradiation can increase the efficiency and flexibility of stress response networks [26,28,37,38]. Moreover, it has been previously reported that the same soybean mutant lines (SM1 and SM3-1) exhibited a more pronounced repertoire of responses under stress [39]. Studies in soybeans suggest that mutant lines harbor a greater number of genome-wide DNA polymorphisms, leading to a higher frequency of DEGs under stress conditions. This increase is generally attributed to mutations within regulatory regions of the genome [34,40]. Consistently, Meriç et al. (2023) reported extensive re-arrangements in the proteome of gamma-induced soybean mutants compared to their parental counterparts [30].

Generated Venn diagrams revealed common gene clusters of 461 upregulated genes and 407 downregulated genes in the mutant lines compared to the parental S04-05 line under both NaCl and PEG treatments (Figure 1C,D). These data demonstrate that the soybean possesses a conserved core stress response mechanism that is activated irrespective of the stress type (salt or drought) or genotype (SM1, SM3-1). These DEGs are indicative of the plant’s cross-tolerance strategy against osmotic imbalance, a primary component of both stresses. Studies indicate that such core genes typically encode essential protective proteins, including transcription factors that regulate antioxidant defenses, ion balance, and cellular homeostasis [36,41]. Although more extensive gene activation was observed in SM1 and SM3-1 mutants, the conservation of 868 core genes indicates that gamma-induced mutagenesis did not disrupt the core stress response framework critical to plant survival. Instead, it is thought that successive selection processes under drought and salinity conditions favored individuals maintaining this conserved core structure and additionally activated regulatory and metabolic components that enhanced adaptive responses.

According to GO enrichment analyses, soybean mutant lines showed significantly different gene expression patterns compared to the parental line under both NaCl and PEG treatments (Figure 3). This indicates that stress tolerance in mutants is regulated by different biochemical and physiological mechanisms. Proteomic studies performed on the lines improved by mutation breeding have reported mutant-specific changes in the abundance of proteins associated with many BPs, such as photosynthesis, carbon metabolism, redox regulation, and membrane stability [30,39]. Similarly, proteomic analyses performed on drought-tolerant tomato mutants improved by gamma radiation revealed significant re-arrangements in ATP and carbohydrate metabolism, mRNA processing and protein phosphorylation, oxidation reduction and stress response, signaling and the supporting cytoskeleton [42]. In the current study, the SM3-1 mutant subjected to NaCl treatment presented more extensive transcriptional regulation in BPs related to photosynthesis, oxidative stress response, hormone signaling, and transmembrane transport compared to the parental line. These findings are consistent with existing knowledge that photosynthetic capacities and antioxidant/oxidative stress pathways are enhanced in salinity-tolerant soybean genotypes, although a decrease in chlorophyll content and inhibition of photosynthesis were generally observed against salt stress. Comparative transcriptome analyses concluded that photosynthetic activity was higher in salinity-tolerant soybeans than in salt-sensitive ones [35]. Furthermore, it has been reported that salt stress significantly enriches pathways such as phenylpropanoid biosynthesis, which plays a critical role in maintaining ROS (reactive oxygen species) detoxification and intracellular balance, and that these pathways are specifically enriched in salinity-tolerant soybean [35,43]. Comparative transcriptomic studies between salt-tolerant and sensitive varieties have identified 3588 DEGs associated with salt tolerance, predominantly involved in ion transport, oxidative stress, and photosynthesis [44]. In this study, the extensive transcriptomic response found in the SM3-1 mutant suggests a multifaceted adaptation strategy involving photosynthesis and oxidative stress pathways under stress. Studies have reported that the maintenance of chloroplast functions and the re-arrangement of genes associated with photosynthetic membranes play a critical role in stress adaptation in salt-tolerant plant genotypes [45].

The enrichment of BPs associated with defense response, chemical stimulus response, and ion transport in the SM1 mutant under NaCl treatment demonstrates that this mutant activates adaptation strategies to environmental salt stress. Pruthi et al. (2025) [46] compared transcriptomic responses of salinity-tolerant (PI 561363) and -sensitive genotypes (PI 601984), and GO analysis revealed enrichment in processes such as ion transport, ethylene signaling, suberin biosynthesis, lipid biosynthesis, and metabolism. Furthermore, the tolerant soybean genotype exhibited higher chlorophyll content and lower malondialdehyde (MDA) and peroxidase (POX) activity compared to the sensitive genotype under salt stress. Additionally, significant enrichment was reported in the categories of ion homeostasis, phosphorylation, and carbohydrate metabolism, which are essential for the Na^+^ and Cl^−^ ion regulations and the mitigation of osmotic stress [46]. Salt stress induces physiological effects, including oxidative stress and membrane lipid peroxidation in soybeans. These findings underscore the critical role of transporter and signaling mechanisms in maintaining intracellular structures. Various studies in soybeans have shown that salt stress significantly regulates transporter and ion homeostasis pathways [43].

The enrichment of BPs associated with photosynthesis, energy metabolism, and phosphorylation in the SM3-1 mutant subjected to PEG treatment indicates that this line initiates molecular regulation, particularly in chloroplast and plastid structures, to counteract oxidative stress. Various transcriptomic analyses have shown that photosynthesis is inhibited, and genes related to photosystem components are regulated in plants under PEG-simulated drought stress [19].

The enrichment of BPs related to hormone response and carbohydrate metabolism in the SM1 mutant under PEG treatment suggests that plant responses to water deficiency are effective for cellular signal transduction and maintaining growth and development. Under drought stress conditions, signal transduction and plant hormones, particularly abscisic acid (ABA), play a vital role in soybean drought tolerance [47]. Regulation of carbon metabolism pathways such as pentose–phosphate, glycolysis, and the TCA (tricarboxylic acid) cycle aims to maintain energy balance and support stress tolerance [48,49].

Genotype and stress condition-specific KEGG pathway enrichment analyses revealed shared and divergent stress response mechanisms of mutant lines, enabling the evaluation of different adaptation strategies (Figure 4). The shared enrichment of secondary metabolite and isoflavonoid biosynthesis pathways in SM3-1 and SM1 mutants under NaCl stress suggests that mutagenesis constitutes a key defense mechanism in these lines, maintaining cellular redox balance and optimizing the oxidative stress response. The functional divergence between the lines reveals differing stress adaptation strategies. The shared enrichment of the plant hormone signal transduction pathway by the SM3-1 mutant under both NaCl and PEG treatments indicates that this line proactively coordinates its response to environmental changes via hormonal signaling networks. Specifically, the marked enrichment of pathways related to photosynthesis and antenna proteins by the SM3-1 mutant under NaCl treatment demonstrates that hormonal regulation plays a strategic role in maintaining the structural integrity and photochemical activity of the photosynthetic apparatus. These findings are consistent with the knowledge [44] that hormonal signaling mechanisms enhance stress tolerance by balancing chloroplast homeostasis. In contrast, the SM1 mutant, unlike SM3-1, developed a more metabolite-based osmotic protection and ROS scavenging strategy, focusing on flavonoid biosynthesis under NaCl stress, starch and sucrose metabolism, amino sugar and nucleotide sugar metabolism, and isoflavonoid biosynthesis pathways under PEG treatment. The osmotic stabilizing roles of flavonoids and sugars are identified as critical “metabolic checkpoints” for stress adaptation in soybeans [39,50]. These findings collectively suggest that the dynamic response of the SM3-1 mutants based on hormone–photosynthesis interactions and the structural protection strategy of the SM1 mutants based on flavonoid and carbohydrate metabolism reflect the generation of diverse genetic resources utilizing different physiological mechanisms while exhibiting similar levels of tolerance through mutagenesis.

KEGG enrichment analyses also accentuate that the SM1 and SM3-1 mutant lines have developed different but effective molecular adaptation strategies against salinity and PEG-simulated drought stress (Figure 5). The prominence of metabolic pathways, photosynthesis, and isoflavonoid biosynthesis in both mutant lines under NaCl treatment suggests that energy metabolism and antioxidant defense are co-regulated under salt stress. Recent studies revealed that maintaining photosynthesis and flavonoid/isoflavonoid accumulation play a critical role in salt tolerance [22]. The prominence of the fatty acid degradation pathway under NaCl treatment in the SM1 mutant suggests that this line has developed a metabolic strategy that supports energy production and membrane remodeling under stress conditions. The importance of lipid metabolism in adaptation to salinity and drought stresses is emphasized, particularly by recent transcriptomic studies [35,46]. The enrichment of glyoxylate and dicarboxylate metabolism in both mutant lines under PEG treatment indicates that carbon metabolism has been redirected to adapt to stress conditions. Recent studies have clearly shown that the glyoxylate cycle increases carbon efficiency and supports growth under drought stress [51]. The prominence of phenylpropanoid biosynthesis in the SM3-1 mutant suggests that this line has developed an adaptation to enhance cell wall fortification and antioxidant capacity. In contrast, the simultaneous activation of photosynthesis and lipid metabolism in the SM1 mutant points to a stress response based on the maintenance of chloroplast integrity. Direct comparison of both stress conditions (NaCl and PEG) revealed limited pathway differentiation between mutant lines. This suggests that SM1 and SM3-1 mutants respond to different abiotic stressors by activating largely common molecular pathways. However, genotype-specific differences in some pathways are noteworthy depending on the type of stress. In this context, the enrichment of only the plant hormone signaling pathway in the SM3-1 mutant indicates that this line is more dependent on hormonal regulation in stress perception and response processes [52]. Hormone signaling, particularly through ABA and jasmonate (JA) pathways, plays a central role in the regulation of stress tolerance and is well-established in the literature [53,54]. In contrast, the enrichment of glyoxylate metabolism, plant–pathogen interaction, and general metabolic pathways in the SM1 mutant suggests that this genotype activates metabolic reprogramming and defense-related processes, selectively, under different stress conditions [55].

Under physiological growth conditions, SM3-1 and SM1 mutants exhibited increased gene expression of the isoflavonoid biosynthesis pathway (Figure 6). Gamma-induced mutants of this study were developed by deliberate in vivo and in vitro selections specifically targeting salinity and drought tolerance in each generation [13]. KEGG enrichment analysis revealed that, consistent with their selected stress-tolerant phenotypes, the mutant lines exhibited higher expression of genes associated with secondary metabolite pathways even under untreated conditions. Therefore, it was expected that the selection process would yield mutant lines with higher isoflavonoid content [56], as these metabolites are key indicators of enhanced stress resilience. Flavonoids play critical roles in legumes such as soybeans, by means of antioxidant defense, cell wall stability, and stress signaling. As stress-responsive metabolites, flavonoids play a significant role in mitigating oxidative damage caused by abiotic stress by scavenging various ROS [57]. Previous studies have shown that abiotic stress increases flavonoid accumulation in various plant species [18]. Wang et al. (2024) [21] reported that total flavonoid content was higher in salt-tolerant plants than in salt-sensitive plants following salt stress treatment, suggesting that these plants possess a stronger ROS-scavenging capacity, thereby reducing ROS-induced cellular damage under salt stress conditions. Flavonoids are not only antioxidant compounds that scavenge ROS as stress-responsive secondary metabolites but also important regulators contributing to the coordination of cellular structure and signaling. Increased ROS production under abiotic stress conditions leads to oxidative damage in cells and also acts as an important second messenger in abiotic stress signaling. In this context, flavonoid accumulation limits oxidative damage by maintaining ROS levels in balance and contributes to the precise regulation of ROS-mediated signaling pathways [58]. Indeed, the increased ROS scavenging capacity of flavonoids enhances the effectiveness of adaptive responses by protecting the cellular integrity of plants under stress conditions. In addition, flavonoids protect the stability of cell membranes by reducing lipid peroxidation and indirectly contribute to the maintenance of cell wall integrity. Maintaining cell wall stability is critical for maintaining cell turgor and mechanical strength, especially under salt and drought stress. Since structural changes in the cell wall also play a role in the sensing and transmission of stress signals, the modulation of these processes by flavonoids via ROS establishes a significant link between cell wall integrity and signal transduction [59]. This demonstrates that flavonoid metabolism is not merely a defense mechanism but an integrated system that acts as a bridge between cellular structure and signaling networks.

High accumulation of these compounds can increase metabolic flexibility under both stress and non-stress conditions. Numerous studies have shown that elevated levels of flavonoids under stress conditions, including flavones, flavanols, flavanones, flavanols, flavanonols, isoflavones, chalcones and anthocyanins, enhance the response time and effectiveness of plant stress responses [18,23]. Extensive transcriptomic and metabolomic analyses performed after PEG-simulated drought stress revealed that metabolic regulation, including flavonoid and isoflavone pathways, plays a significant role in the response of soybean plants to drought stress and that isoflavone metabolites and related genes are involved in the process [19]. Specifically, the increased expression of the isoflavonoid pathway under non-stress conditions suggests that mutant lines may possess a constitutively active secondary metabolism, reflecting a possible metabolic “priming” state. Such basal activation could allow plants to respond more quickly and effectively to subsequent stress conditions by maintaining key defense-related pathways in a ready state. This perspective is important for understanding how mutation-induced changes alter metabolic balance not only under stress conditions but also under normal growth conditions. Supporting this view, previous studies have shown significant variation in the accumulation of antioxidant compounds among gamma-irradiated soybean mutant lines; different genotypes with high anthocyanin, epicatechin, or isoflavone content emerged. This indicates that mutation breeding can lead to pronounced metabolic reprogramming across different lines [56].

DEG analysis revealed that several key enzymes involved in the isoflavonoid biosynthesis pathway were differentially expressed under NaCl stress. Among these, the expression of the flavone synthase IIs ([EC:1.14.19.76], Glyma.12067000) gene was not regulated in the SM3-1 mutant but decreased in the SM1 compared to S04-05. Obviously, the enzyme that catalyzes the conversion of flavanones to flavones by introducing a double bond between C-2 and C-3 through an oxidative reaction [60] exhibits different expressions in different mutant genotypes developed through mutation breeding, leading to differences in metabolic flow. Plants produce specific flavonoid derivatives by diversifying the flavonoid skeleton through successive modifications such as hydroxylation, *O*-methylation/*O*-demethylation, and acylation [61]. The upregulation of the genes related to hydroxylation, *O*-methylation, and acylation steps involved in the isoflavonoid biosynthesis pathway under stress conditions indicates that isoflavonoid metabolism is redirected toward the production of more stable and defense-related derivatives. Previous reports pointed to genes in the flavonoid/isoflavone pathway and showed different modulations during salinity stress depending on the genotypes [21]. This supports the assertion that mutant lines respond to NaCl treatment with different metabolic strategies. It has been reported that increased accumulation of flavonoids and isoflavones in soybeans strengthens the defense against ROS and increases stress tolerance [18,19,21,22].

DEG analysis indicated that PEG treatment caused complex regulation of the isoflavonoid pathway in both SM3-1 and SM1 mutants. In SM3-1, the downregulation of genes such as isoflavone 4′-O-methyltransferase ([EC:2.1.1.212 2.1.1.46], Gly-ma.13G173300 and Glyma.13G173600) and 2′-hydroxyisoflavone reductase ([EC:1.3.1.45], Glyma.01G211800 and Gly-ma.11G070500), while 28 genes of other enzymatic steps were upregulated, indicates a dynamic rearrangement of the pathway in the SM3-1 mutant. Similarly, in SM1, decreased expression of flavone synthase IIs ([EC:1.14.19.76], Glyma.12G067000), isoflavone 2′-hydroxylase ([EC:1.14.14.90 1.14.14.89], Glyma.09G048700) and pterocarpan synthase ([EC:42.1.139], Glyma.19G151100) enzymatic steps and increased expression in 26 other genes suggest that drought stress in the SM1 mutant utilize a regulatory network targeting different steps of the pathway. This finding is consistent with broader omics analyses showing that flavonoid and isoflavonoid pathways are not entirely upregulated or downregulated under drought stress but rather that metabolic flow is partly regulated in terms of energy and resource utilization [19]. Studies revealing the association of flavonoid metabolite levels, along with isoflavonoids, with drought tolerance under PEG stress highlight that molecular plasticity in these pathways is key to stress tolerance [19]. Ackah et al. (2021) [62] reported that polyphenols, primarily antioxidant secondary metabolites including flavonoids and cinnamic acids, undergo a dynamic change in the plant under drought stress, showing both increasing and decreasing patterns. The accumulation or decrease in flavonoids under drought stress has been reported as an ordinary response of plants due to factors such as treatment time, species, and degree of drought stress [19]. Therefore, different genotypes of soybeans exhibit different responses to drought stress. In another study conducted with drought-stressed soybean plants, it was reported that four flavonoids accumulated in the drought-resistant soybean variety Heinong 44 (HN44) and seven flavonoids accumulated in the drought-sensitive soybean variety Heinong 65 (HN65) [63].

Detailed examination of gene expression within the isoflavone biosynthesis pathway revealed significant differences in stress response among genotypes (Figure 7). The suppression of the vast majority of genes in the pathway under both NaCl and PEG treatments in the parental S04-05 line indicates a limited secondary metabolism-based defense capacity in this genotype. In contrast, a widespread upregulation in gene expression across the pathway under NaCl and PEG treatments, particularly in the SM3-1 mutant, suggests that isoflavone biosynthesis is actively involved in the stress response. Overall, the coordinated upregulation of numerous isoflavone pathway genes in the mutant lines demonstrates that this metabolic pathway is a critical component of tolerance to both salinity and drought stresses. Recent studies have shown that flavonoid and isoflavonoid compounds play a central role in ROS detoxification, osmotic balance, and cellular protection mechanisms, particularly under drought and salinity stress [64,65,66,67]. Flavonoids are essential secondary metabolites that play a critical role in stress mitigation due to their antioxidant properties, which reduce oxidative stress by scavenging excess ROS, thus protecting plants from growth inhibition and cell death [68]. Similarly, transcriptomic and metabolomic studies have reported that isoflavonoid pathway genes and related metabolites are increased concomitantly under drought stress, and this increase is directly associated with stress tolerance [64,69].

SNP analysis revealed thousands of variants unique to the mutant lines. The presence of 10,070 and 6418 unique SNPs in the SM1 and SM3-1 mutants, respectively, as well as approximately 12,071 shared SNPs, suggests that gamma mutagenesis leads to novel variations on a large scale (Figure 8). This is consistent with the fact that irradiation is known to cause double-strand breaks and widespread structural changes in DNA, which in turn lead to numerous SNPs and indels in plants [70]. Meriç et al. (2023) demonstrated that similar genomic changes underlie the extensive proteome differences between the mutant lines and the parental line [30]. Therefore, our SNP analysis points to a possible genetic basis for the observed gene expression differences and suggests that different molecular strategies for stress tolerance in mutant lines may be related to genomic diversity; however, further functional analyses are required to determine the direct effects of these variations on gene expression or protein function. Comparing the SM1 and SM3-1 mutants, the higher total number of SNPs in the SM1 mutants indicates a broader mutation load. Additionally, the presence of 10,070 unique SNPs in the SM1 mutant and 6418 in the SM3-1 mutant reveals the extent of gamma radiation and associated ROS activity in causing single-nucleotide polymorphisms.

Comparative SNP analyses suggest that variations concentrated in genes associated with the isoflavonoid biosynthesis pathway in SM3-1 and SM1 mutant lines may contribute to fine-tuning and regulatory changes rather than completely disrupting pathway function (Table 1). The predominance of moderate-impact mutations, such as missense variants, over high-impact mutations (e.g., frameshift, stop-gain, or splice-site) suggests that gamma mutagenesis in this study primarily modulates gene expression levels and metabolic flux rather than disrupting essential protein functions [71,72]. The fact that the SNPs identified in the isoflavonoid and flavonoid metabolism-associated enzymes, such as flavonoid 6-hydroxylase (*F6H*, Glyma.11G108300), 2-hydroxyisoflavanone synthase (*IFS2*, Glyma.13G173500) and isoflavone 7-O-glucosyltransferase (*UGT7*, Glyma.16G175900), as well as regulatory proteins such as MYB transcription factor (*GmMYB176*, Glyma.05G032200) and NmrA-like family domain-containing protein (*IFR4*, Glyma.11G070600), are categorized as moderate-impact or modifier SNPs indicates that the essential catalytic functions of these proteins are likely preserved. On the other hand, it suggests that the genes in the pathway may have been rearranged in terms of expression levels and timing [73]. Similarly, variations in genes involved in flavonoid and isoflavonoid biosynthesis have been reported to play a role in metabolic regulation under stress conditions [74]. Another notable finding is the presence of multiple SNPs in MYB transcription factors in both mutants. The 3′UTR variations and missense mutations found in MYB transcription factors, which are recognized as important regulators of isoflavone and flavonoid biosynthesis, may be a factor in the varying expression patterns of pathway genes under stress [75]. In this study, homologous protein models constructed for genes involved in the isoflavone biosynthesis pathway revealed that SNP-induced variants did not lead to significant conformational changes in the three-dimensional structures of the proteins (Figure 10). The high structural similarity between mutant and parental line proteins suggests that these variants are tolerable in terms of protein folding and overall structural integrity. However, the absence of noticeable structural changes does not preclude potential functional effects, as variants may still influence protein stability, regulatory dynamics, or molecular interactions [76]. This finding is consistent with the literature showing that genetic variations contributing to stress tolerance in plants do not always lead to dramatic changes in protein structure; instead, they can exert functional effects through other molecular mechanisms such as gene expression levels, metabolic flow, or regulatory networks. For example, comprehensive structural analyses have revealed that single amino acid substitutions can exert differential effects on stability and function across all protein classes and that these effects vary depending on position, structural environment, and functional context [77,78]. Furthermore, the functional effects of protein variants can influence not only structural stability but also broader molecular properties such as catalytic activity, interaction surfaces, and regulatory functions.

In conclusion, these findings suggest that in SM3-1 and SM1 mutant lines developed by gamma mutagenesis, isoflavonoid biosynthesis may play a role in tolerance to stress conditions by maintaining gene expression in the pathway under the influence of low- and medium-impact variations, rather than high-impact disruptive mutations. The dynamic regulation of flavonoid and isoflavonoid biosynthetic pathways is closely related to plant responses to abiotic stresses such as salinity and drought, and these metabolic pathways are thought to play a role in stress adaptation.

## 4. Materials and Methods

### 4.1. Plant Material and Growth Conditions

The present study used salt- and drought-tolerant M_4_ generation SM1 and SM3-1 soybean mutant lines that were previously developed by Atak et al. (2024) [13] and their original S04-05 soybean variety. Mutants were developed by Cs-137 gamma irradiation of the S04-05 variety. The initial M_1_ generation was established through single-plant harvesting. Importantly, the SM3-1 and SM1 mutants used in this study originated from the same M_1_ plant (code: 150-106) and were subsequently advanced through the M_2_, M_3_, and M_4_ generations with in vivo and in vitro selection processes [13]. M_4_ generation plants were used for transcriptomic analysis. Plants were cultivated in the Plant Biotechnology Laboratory of Istanbul Kultur University, Turkey. Plants were germinated in Murashige and Skoog (MS) (Duchefa Biochemie B.V., Haarlem BH, The Netherlands) for 7 days. Subsequently, the stress groups were transferred to MS medium containing 90 mM NaCl and 10% polyethylene glycol 6000 (PEG) and grown at 25 °C under a 16/8 light/dark period for 14 days. The experiment was designed as nine experimental groups using three soybean lines (SM1, SM3-1, and parental S04-05) under three conditions (untreated, NaCl, and PEG). Each group consisted of three biological replicates (60 plants per replicate). Ten plants from each biological replicate were pooled for RNA extraction, and all analyses were performed in three technical replicates for each biological sample. The mutation breeding methodology and the physiological responses of soybean mutants derived from the same harvest period to stress factors, including proline accumulation, soluble sugar levels, and photosynthetic pigment content, were described in detail in our previous publication [13].

### 4.2. RNA Extraction and Quality Control

Total RNA was extracted from the collected leaf samples by using TRIzol (Invitrogen, Thermo Fisher Scientific, Carlsbad, CA, USA) according to the manufacturer’s instructions. RNA quality and quantity were assessed by using a NP80 nanodrop (Implen GmbH, Munich, Germany) and Agilent 2100 Bioanalyzer (Agilent Technologies, Santa Clara, CA, USA). Only RNA samples with RNA integrity number (RIN) values greater than 7.0 were used for library preparation.

### 4.3. RNA-Seq Library Preparation and Sequencing

Total RNA samples were processed by using the TruSeq RNA Sample Preparation Kit (Illumina, San Diego, CA, USA) to generate Pol(A) + libraries, with Pol(A) + mRNA isolated from total RNA using magnetic beads according to the manufacturer’s instructions, followed by fragmentation, reverse transcription, second strand synthesis, end-repair, A-tailing, adapter ligation, and PCR amplification. Library quality and quantity were assessed using the Agilent 2100 Bioanalyzer (Agilent Technologies, Santa Clara, CA, USA) and Qubit 2.0 Fluorometer (Life Technologies, Carlsbad, CA, USA). Prepared libraries were sequenced on the Illumina NovaSeq 6000 platform (Illumina Way, San Diego, CA, USA) as 2X 150 bp 15–20 million paired-end reads (a total of ~30–40 million reads). Illumina sequencing raw data were deposited in the NCBI SRA database under the accession number PRJNA1393611.

### 4.4. Bioinformatics Analysis

#### 4.4.1. Quality Control and Pre-Processing of Raw Reading Data

The quality of raw RNA-seq read data was determined by using FastQC (v0.11.9) software. Low-quality (phred score < 25), short reads (<40 bp) and adapter sequences were cleaned by using Fastp (v1.0.1) [79].

#### 4.4.2. Reference Genome Alignment, Identification of Expression Differences and Enriched Pathways

The trimmed read data were aligned to the reference genome (Glycine_max_Wm82.a2.v1) by using the STAR (Spliced Transcripts Alignment to a Reference v2.7.11a) alignment tool. The resulting BAM files were sorted and indexed using Samtools (version 1.22.1). Only properly paired and uniquely mapped reads were used. Raw counts for genes were counted using the featureCounts (Subread v2.0.3) tool.

The resulting count matrix was uploaded to the Galaxy platform (https://usegalaxy.eu/, accessed on 2 December 2025). It was organized for use in differential gene expression analysis and performed using the DESeq2 tool (Galaxy Version 2.11.40.8 + galaxy0) with size-factor normalization [80]. Genes with low expression that did not meet the condition of CPM ≥ 1 in at least two samples were eliminated from the analysis. The Benjamini–Hochberg method-corrected *p*-value (FDR < 0.05) was used to identify DEGs, and the threshold |log2 (fold change) ≥ 1 was used for biological significance. Venn diagrams were created using DEG numbers [81].

Transcripts were annotated using the soybean (*Glycine max* Wm82.a2.v1) genome annotation file downloaded from the Phytozome v.14 portal (Joint Genome Institute; https://phytozome.jgi.doe.gov, accessed on 2 December 2025) and matched to transcripts defined by the soybean (*Glycine max* Wm82.a2.v1) genome annotation file using the Annotate DESeq2/DEXSeq output tables (Galaxy Version 1.1.0 + galaxy1) tool. The ShinyGO toolkit was applied for GO and KEGG functional enrichment (FDR < 0.05) analyses [82]. Comparative pathway analyses were performed using the Pathview R package (v4.3.1), with gene colorings on the map based on DEGs [83].

#### 4.4.3. SNP Detection and Filtering

GATK (Genome Analysis Toolkit, v4.6.2.0) software was used for variant detection from RNA-seq data. PCR duplicates were marked in BAM files aligned to the reference genome using STAR and Picard MarkDuplicates (v3.4.0), then RNA-seq-specific splice junctions were corrected using the GATK SplitNCigarReads tool. The GATK HaplotypeCaller module was run for variant calling. Variants were filtered by using the GATK VariantFiltration module: mapping quality (MAPQ) ≥ 20, base quality (Base Quality) ≥ 30, depth of coverage (DP) ≥ 10, Fisher Strand bias (FS) < 30.0, Mapping Quality RankSum (MQRankSum) > −12.5, and Read Position RankSum (ReadPosRankSum) > −8.0. Variants that failed to pass the filters were excluded. First, all sample VCF files were generated according to the Williams 82 reference genome sequence. To eliminate natural polymorphisms between S04-05 and the Williams 82 reference genome and to identify only *de novo* mutations (SM1 and SM3-1 specific SNPs) resulting from gamma irradiation, all variants detected in S04-05 were extracted from the mutant lines’ VCF files using bcftools (v1.23).

#### 4.4.4. SNP Annotation and Functional Characterization

Filtered variants were annotated against the soybean reference genome (Glycine_max_Wm82.a2.v1) by using the Ensembl Variant Effect Predictor (https://plants.ensembl.org/Glycine_max/Tools/VEP, accessed on 2 December 2025) software. The regions of the genome where the SNPs are located (exon, intron, 5′ UTR, 3′ UTR, upstream/downstream gene regions) have been determined. SNPs have been classified as synonymous, missense, nonsense, frameshift, or splice site variants according to their effects. Variant results were categorized using effect categories (HIGH, MODERATE, LOW, and MODIFIER) provided by the Ensembl Plants Variant Effect Predictor (VEP) [84]. LOW effect variants, including synonymous substitutions, were excluded from the analysis. Medium effect variants (e.g., missense, in-frame indels) and modifier variants (e.g., intronic, intergenic) were preferred for their potential functional and regulatory significance. Protein structures predicted by the UniProt database and AlphaFold were used for functional domain annotations and structural evaluations of the potential products of the transcripts. Statistical analyses were performed using GraphPad Prism (v. 9.5.1) and visualizations were performed using the ggplot2 (v.3.4.2) package in R (v4.3.1) software and the Matplotlib library (v3.6.2) in Python (v3.10.16).

#### 4.4.5. Transcription Factor Binding Site Analysis

TFBS prediction analysis was performed on isoflavonoid biosynthesis genes containing SNPs. For each gene, 30 bp sequences of genomic regions containing the SNP position were obtained from the Phytosome v13 database in FASTA format. Analysis was performed using the PWM scanning method (*p* < 0.001) of the PlantPAN 4.0 tool (https://plantpan.itps.ncku.edu.tw/plantpan4, accessed on 2 December 2025) and the results with a similarity score > 0.80 were retained [85].

#### 4.4.6. Conservation Analysis and Homology Modelling

BLASTp analysis was performed using the target protein sequences (NP_001236048.2, NP_001237536.2, XP_003537814.1, NP_001304487.1, NP_001238515) against Fabaceae protein sequences. Homologous sequences were filtered by applying a percent-identity threshold of >60%, and in addition, *Arabidopsis thaliana* and *Oryza sativa* sequences were added. Multiple sequence alignments (MSAs) were generated using the ClustalW algorithm implemented in the msa R package (v4.5.2) and visualized using ggmsa (v1.16.0). Amino acid conservation at specific positions of interest was quantified by calculating frequency distributions and Shannon entropy values, where higher entropy indicates greater variation and consequently lower conservation.

SWISS-MODEL (https://swissmodel.expasy.org/, accessed on 2 December 2025) with automated template selection was used to predict the three-dimensional structures of reference and SNP-derived mutant proteins [86]. For the MYB133, F6H, MYB176, IFS2 and UGT7 proteins, the template with the highest ranking based on sequence identity, coverage, and GMQE (Global Model Quality Estimation) score was selected. Both the reference and mutant proteins were modeled using the same template. SWISS-MODEL output metrics, which are provided in Supplementary S5, were used to evaluate the model’s quality. The resulting structural models were visualized and compared using UCSF ChimeraX (v1.11.1) [87].

## 5. Conclusions

This study suggests that gamma irradiation-induced SM1 and SM3-1 soybean mutants develop different but functional molecular adaptation strategies to salinity and drought stresses. Genotype- (S04-05, SM1, SM3-1) and stress condition (salt or drought)-specific KEGG pathway enrichment analyses showed that the SM1 and SM3-1 mutant lines, originating from the same M1 plant and developed through in vivo and in vitro selection processes, possess both shared and divergent stress response mechanisms, thus revealing different adaptation strategies. The shared enrichment of secondary metabolites, and especially isoflavonoid biosynthesis pathways, in both mutant lines reveals that a fundamental defense mechanism maintaining cellular redox balance and optimizing the oxidative stress response has developed in these lines as a result of mutagenesis. However, the functional divergence between the lines clearly demonstrates the adoption of different molecular strategies in stress adaptation. The SM3-1 line exhibited a structure that proactively coordinates its response to environmental changes through hormonal regulatory networks by consistently enriching plant hormone signaling pathways under both salinity and PEG-simulated drought conditions. The significant enrichment of pathways associated with photosynthesis and antenna proteins, particularly under NaCl conditions, demonstrates that hormonal signaling plays a strategic role in maintaining the structural integrity and photochemical activity of the photosynthetic apparatus and contributes to the sustainability of chloroplast homeostasis. In contrast, the SM1 mutant line developed a more metabolite-based osmotic balance and ROS scavenging strategy, focusing on pathways such as flavonoid and isoflavonoid biosynthesis, starch and sucrose metabolism, and amino sugar and nucleotide sugar metabolism. In this context, it is understood that flavonoids and soluble sugars function as critical “metabolic checkpoints” in stress adaptation in soybeans. Furthermore, the prominence of the fatty acid degradation pathway under NaCl stress in the SM1 line supports the existence of metabolic adaptation based on energy production and membrane remodeling, while the enrichment of glyoxylate and dicarboxylate metabolism observed in both mutants under PEG conditions indicates that carbon metabolism has been redirected to adapt to stress conditions. The prominence of phenylpropanoid biosynthesis in the SM3-1 line indicates an adaptation toward cell wall strengthening and increased antioxidant capacity. Although the two mutant lines respond to different abiotic stresses by activating largely common molecular pathways, it appears that the SM3-1 line has developed a more hormone-signaling-dependent stress perception and response mechanism, while the SM1 line selectively activates processes more associated with metabolic reprogramming and defense. In this context, the results obtained are of great importance for breeding purposes because the study reveals that tolerance to abiotic stresses such as drought and salinity is not dependent on a single mechanism but can be achieved through different and complementary molecular pathways.

In both mutant lines, the upregulation of numerous genes involved in the isoflavone biosynthesis pathway under salinity, drought and untreated conditions reveals that this pathway plays a key and sustained role in stress tolerance. The vast majority of SNPs identified in the isoflavone biosynthesis pathway consist of moderate-impact and modifier variations. Furthermore, variations detected in MYB transcription factors point to potential regulatory differences that may play a role in genotype-specific regulation of flavonoid and isoflavone pathways. These findings suggest that gamma mutagenesis and subsequent selection processes allow for the development of novel genetic variants that operate through different physiological and metabolic mechanisms but exhibit similar levels of tolerance. In this respect, the study reveals that mutation breeding is a potentially sustainable and effective breeding strategy for increasing abiotic stress tolerance in soybeans.

## Figures and Tables

**Table 1:** Single-nucleotide polymorphisms in isoflavonoid biosynthesis pathway genes from SM1 and SM3-1 mutant lines. Each row represents an SNP with Region (chromosome:position), Reference (nucleotide change: Ref → Alt), Gene_ID (gene identifier/name), Function (enzyme role), SM1 and SM3-1 columns indicating SNP presence (+ = present, − = absent in mutant line), Exon (genomic location: exon, intron, UTR), Impact (predicted functional effect: High/Moderate/Low/Modifier), and Consequence (variant effect term).

Region	Reference	Gene_ID	Function	SM1	SM3-1	Exon	Impact	Consequence
5:2803010	C→A	Glyma.05G032200/*GmMYB17*6	MYB Transcription Factor	+	+	UTR	Modifier	3′UTR Variant
5:2803327	C→T	Glyma.05G032200/*GmMYB17*6	MYB Transcription Factor	+	+	3/3	Moderate	Missense (G232D)
5:2804511	T→G	Glyma.05G032200/*GmMYB17*6	MYB Transcription Factor	+	+	1/3	Moderate	Missense (N46T)
7:5960972	C→T	Glyma.07G066100/*GmMYB13*3	MYB Transcription Factor (*CCA1-like*)	−	+	1/8	Moderate	Missense (A21V)
11:5274437	T→C	Glyma.11G070600/*IFR4*	NmrA-like family domain-containing protein	+	−	−	Modifier	Upstream Gene Variant
11:8262915	T→C	Glyma.11G108300/*F6H*	Flavonoid 6-hydroxylase	−	+	1/2	Moderate	Missense (T248A)
13:28762800	G→T	Glyma.13G173500/IFS2	2-hydroxyisoflavanone synthase	+	+	2/2	Moderate	Missense (K340N)
16:33726259	A→C	Glyma.16G175900/UGT7	Isoflavone 7-O-glucosyltransferase	+	+	1/1	Moderate	Missense (E428D)

**Table 2:** Conservation of missense SNP-derived positions in isoflavonoid biosynthesis pathway proteins. For each position, the most frequent residue, conservation percentage, variant count (i.e., the number of different amino acids at the SNP position), and Shannon entropy values (lower entropy indicates higher conservation) were determined. Conservation percentage reflects the frequency of the dominant residue, while Shannon entropy provides a measure of sequence variability, with lower values indicating higher conservation. Positions with high conservation and low entropy are considered to be under stronger evolutionary constraint and are therefore more likely to be functionally important, as mutations at these sites may disrupt protein function.

Protein/Position	Most Frequent Amino Acid	Conservation Percentage (%)	Variant Count	Entropy
*MYB133*/(21)	G	38.98	6	2.094
*F6H*/(248)	A	51.02	5	1.783
*MYB176*/(46)	N	43.28	9	2.149
*MYB176*/(232)	D	78.95	7	1.238
*IFS2/(340)*	K	90.7	3	0.324
*UGT7/(428)*	E	95.24	3	0.522

## Data Availability

The data that support the findings will be available in NCBI at http://www.ncbi.nlm.nih.gov/bioproject/1393611 (accessed on 25 December 2025) following an embargo from the date of publication to allow for the commercialization of research findings.

## Supplementary Materials

The following supporting information can be downloaded at: https://www.mdpi.com/article/10.3390/plants15081241/s1.

## Abbreviations

The following abbreviations are used in this manuscript:

DEG	Differentially Expressed Gene
SNP	Single Nucleotide Polymorphism
DNA	Deoxyribonucleic Acid
NGS	Next-Generation Sequencing
RNA	Ribonucleic Acid
RNA-seq	RNA Sequencing
PEG	Polyethylene Glycol
GO	Gene Ontology
KEGG	Kyoto Encyclopedia of Genes and Genomes
NCBI	National Center for Biotechnology Information
Mb	Megabase
UDP	Uridine Diphosphate
UTR	Untranslated Region
ExPASy	Expert Protein Analysis System
UniProt	Universal Protein Resource
MYB	MYB Transcription Factor (Myeloblastosis family)
ATP	Adenosine Triphosphate
MDA	Malondialdehyde
POX	Peroxidase
ROS	Reactive Oxygen Species
EC	Enzyme Commission number
MSkoog	Murashige and Skoog medium
PCR	Polymerase Chain Reaction
SRA	Sequence Read Archive
STAR	Spliced Transcripts Alignment to a Reference
BAM	Binary Alignment/Map
CPM	Counts Per Million
FDR	False Discovery Rate
DESeq	Differential Expression Sequencing
GATK	Genome Analysis Toolkit
MAPQ	Mapping Quality
