# Supplementary material for: Comparative Transcriptomic Analysis Reveals Divergent Stress Adaptation Strategies in Gamma-Induced Soybean Mutants
Source: Plants (Basel). 2026 Apr 17;15(8):1241. doi: 10.3390/plants15081241 (PMC13120069; doi:10.3390/plants15081241)
Supplement: Supplementary file 1 [file plants-15-01241-s001.zip › Supplementary S2.pdf]

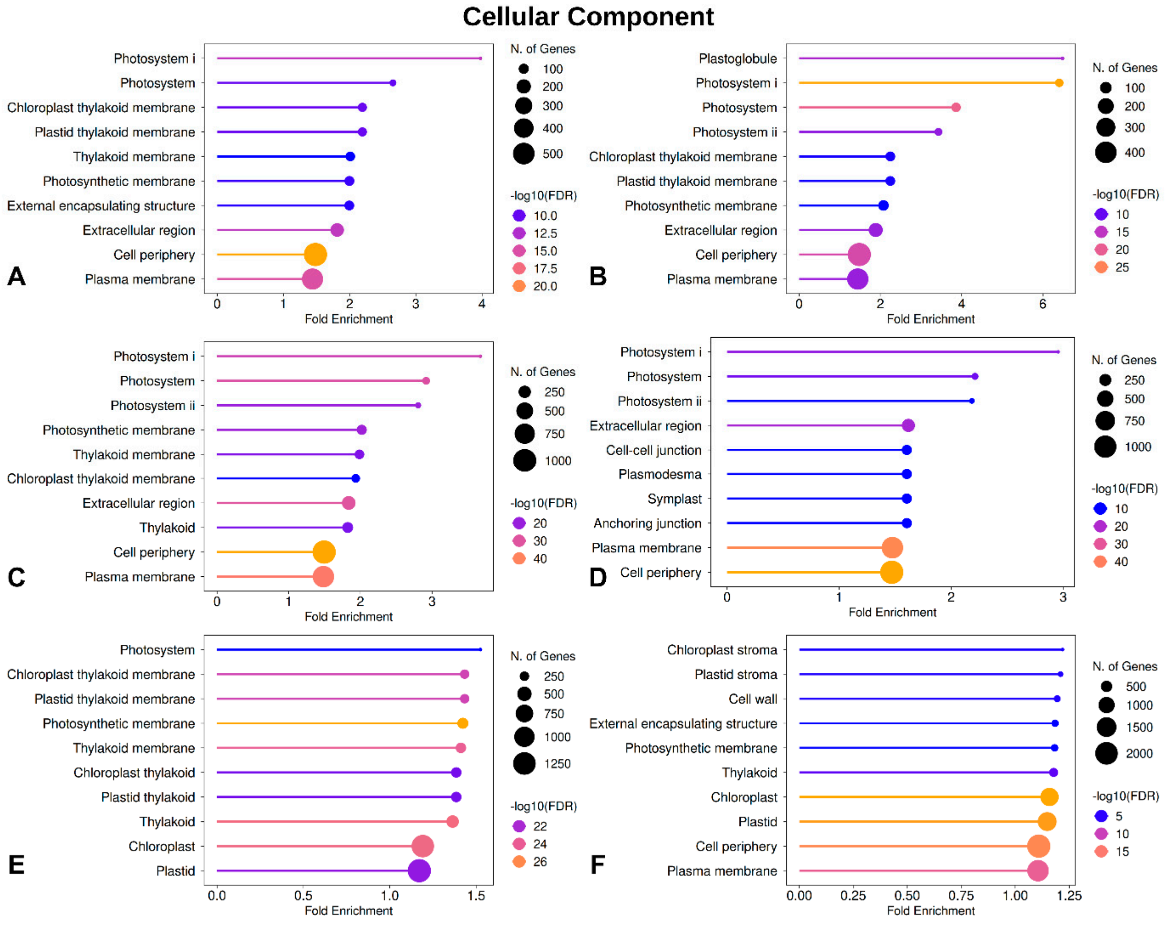


**Figure 3S1.** Cellular Component (CC) Gene Ontology enrichment analysis of genotype and stress condition-related differentially expressed genes. (**A**) SM3-1 control vs. S04-05 control (**B**) SM1 control vs. S04-05 control (**C**) SM3-1 NaCl vs. S04-05 NaCl (**D**) SM1 NaCl vs S04-05 NaCl (**E**) SM3-1 PEG vs. S04-05 PEG (**F**) SM1 PEG vs. S04-05 PEG. Lollipop plots display significantly enriched pathways (FDR < 0.05) with dot size indicating gene counts and color representing -log10(FDR).

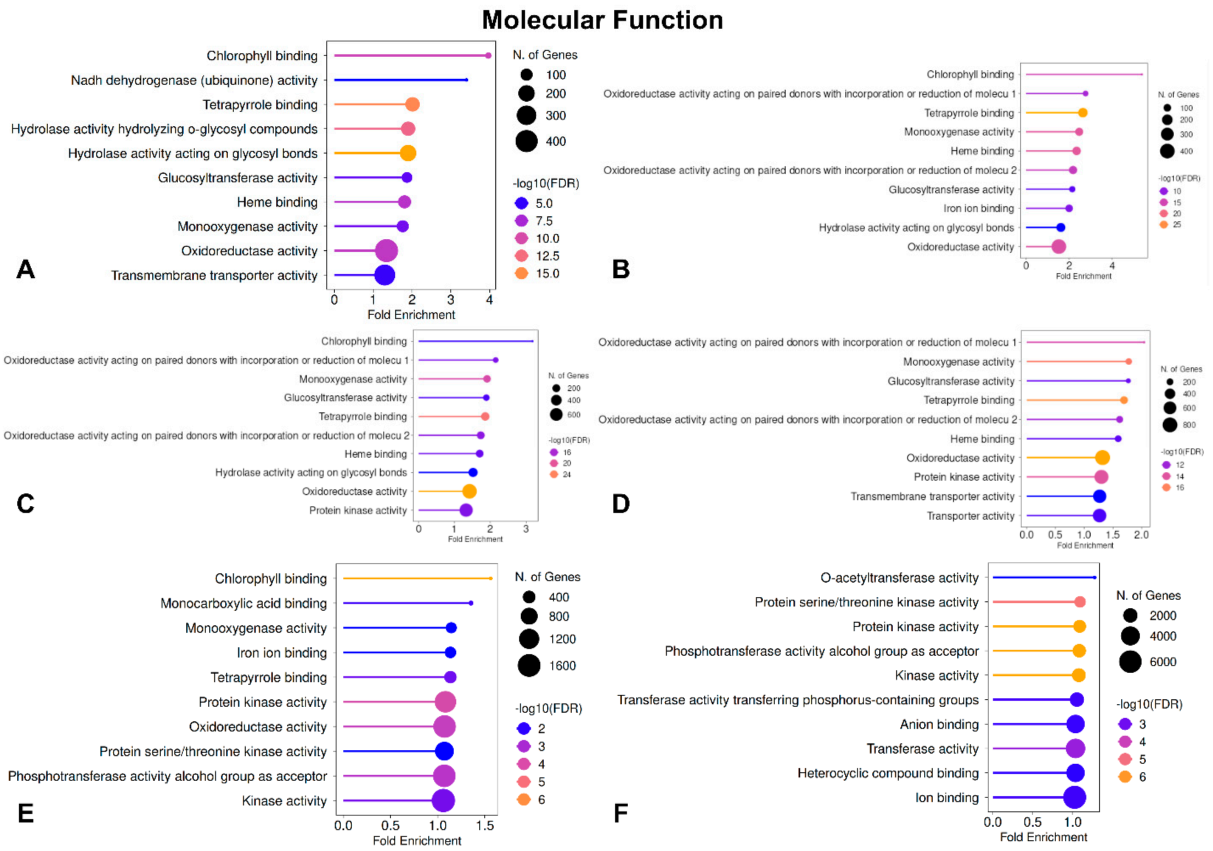


**Figure 3S2.** Molecular Function (MF) Gene Ontology enrichment analysis of genotype and stress condition-related differentially expressed genes. (**A**) SM3-1 control vs. S04-05 control (**B**) SM1 control vs. S04-05 control (**C**) SM3-1 NaCl vs. S04-05 NaCl (**D**) SM1 NaCl vs S04-05 NaCl (**E**) SM3-1 PEG vs. S04-05 PEG (**F**) SM1 PEG vs. S04-05 PEG. Lollipop plots display significantly enriched pathways (FDR < 0.05) with dot size indicating gene counts and color representing -log10(FDR).
