# Supplementary material for: Comparative Transcriptomic Analysis Reveals Divergent Stress Adaptation Strategies in Gamma-Induced Soybean Mutants
Source: Plants (Basel). 2026 Apr 17;15(8):1241. doi: 10.3390/plants15081241 (PMC13120069; doi:10.3390/plants15081241)
Supplement: Supplementary file 1 [file plants-15-01241-s001.zip › Supplementary S4.pdf]

Table S4. Genes containing SNPs and their paralogs

Gene_ID	Gene_Name	Paralog_Gene_ID	Chromosome_Pair	Avg_%Identity	Duplication_Type	Median_Ks
GLYMA_05G032200	GmMYB176	GLYMA_04G177300	Chr05-Chr04	71.8	Whole Genome Duplication (~59 Million Years Ago)	0.714
GLYMA_07G066100	GmMYB133	GLYMA_16G032600	Chr07-Chr16	90.8	Whole Genome Duplication (~13 Million Years Ago)	0.101
GLYMA_11G070600	IFR4	GLYMA_01G172600	Chr11-Chr01	95.9	Whole Genome Duplication (~13 Million Years Ago)	0.136
GLYMA_11G070600	IFR4	GLYMA_11G070500	Chr11-Chr11	85.2	Tandem Duplication	N/A
GLYMA_11G070600	IFR4	GLYMA_01G211800	Chr11-Chr01	85.2	Whole Genome Duplication (~13 Million Years Ago)	0.136
GLYMA_13G173500	IFS2	GLYMA_07G202300	Chr13-Chr07	97.3	Whole Genome Duplication (~13 Million Years Ago)	0.141
GLYMA_11G108300	F6H	No paralog identified	-	-	-	-
GLYMA_16G175800	UGT7	No paralog identified	-	-	-	-
