# Supplementary material for: Comparative Transcriptomic Analysis Reveals Divergent Stress Adaptation Strategies in Gamma-Induced Soybean Mutants
Source: Plants (Basel). 2026 Apr 17;15(8):1241. doi: 10.3390/plants15081241 (PMC13120069; doi:10.3390/plants15081241)
Supplement: Supplementary file 1 [file plants-15-01241-s001.zip › Supplementary S5.pdf]

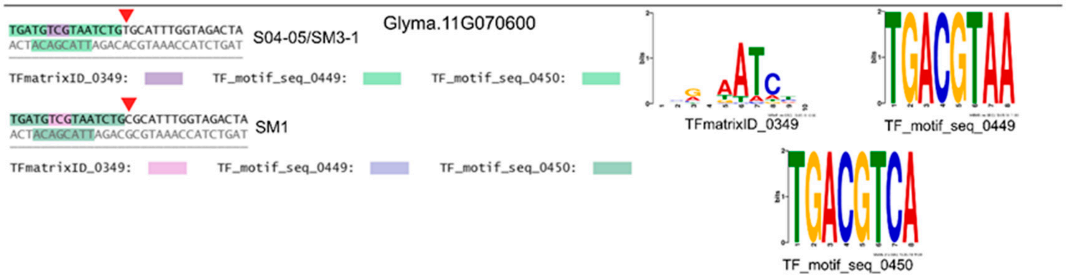

Figure S5.1 Transcription factor binding site (TFBS) analysis of isoflavonoid biosynthesis pathway genes containing SNPs in mutant lines. Colored boxes indicate TFmatrix-based motif predictions, while sequence logos represent the corresponding position weight matrices and consensus motif sequences. Red triangles denote the SNP locations. Glyma.11G070600 (IFR4)

TFmatrixID_0349 / Myb/SANT S04-05

Position	Hit Sequence	Strand	Similar Score
6	tcgtAATCTg	+	1

TF_motif_seq_0449 / (Motif sequence only)

Position	Hit sequence	Strand	Similar score
4	TGTCGtaa	+	0.88

TF_motif_seq_0450 / (Motif sequence only)

Position	Hit sequence	Strand	Similar score
1	TGATGtcg	+	0.75
4	TGTCGtaa	+	0.75
4	tgtCGTAA	-	0.75

TFmatrixID_0349 / Myb/SANT SM3-1

Position	Hit Sequence	Strand	Similar Score
6	tcgtAATCTg	+	1

TF_motif_seq_0449 / (Motif sequence only)

Position	Hit sequence	Strand	Similar score
4	TGTCGtaa	+	0.88

TF_motif_seq_0450 / (Motif sequence only)

Position	Hit sequence	Strand	Similar score
1	TGATGtcg	+	0.75
4	TGTCGtaa	+	0.75
4	tgtCGTAA	-	0.75

TFmatrixID_0349 / Myb/SANT SM1

Position	Hit Sequence	Strand	Similar Score
6	tcgtAATCTg	+	1

TF_motif_seq_0449 / (Motif sequence only)

Position	Hit sequence	Strand	Similar score
4	TGTCGtaa	+	0.88

TF_motif_seq_0450 / (Motif sequence only)

Position	Hit sequence	Strand	Similar score
1	TGATGtcg	+	0.75
4	TGTCGtaa	+	0.75
4	tgtCGTAA	-	0.75

Matrix ID	TF Family	TF ID or Motif Name	Position	Hit Sequence	Strand	Similar Score
TFmatrixID_0349	Myb/SANT	Glyma11g04440.1;Glyma17g16360.1	6	tcgtAATCTg	+	1
TF_motif_seq_0449	(Motif sequence only)	AUXRETGA1GMGH3	4	TGTCGtaa	+	0.88
TF_motif_seq_0450	(Motif sequence only)	PALINDROMICCBOXGM	1	TGATGtcg	+	0.75
TF_motif_seq_0450	(Motif sequence only)	PALINDROMICCBOXGM	4	TGTCGtaa	+	0.75

Figure S5.2 Glyma.11G070600 Gene Pattern Search Results
