# Supplementary material for: Comparative Transcriptomic Analysis Reveals Divergent Stress Adaptation Strategies in Gamma-Induced Soybean Mutants
Source: Plants (Basel). 2026 Apr 17;15(8):1241. doi: 10.3390/plants15081241 (PMC13120069; doi:10.3390/plants15081241)
Supplement: Supplementary file 1 [file plants-15-01241-s001.zip › Supplementary S6.pdf]

Conservation Analysis

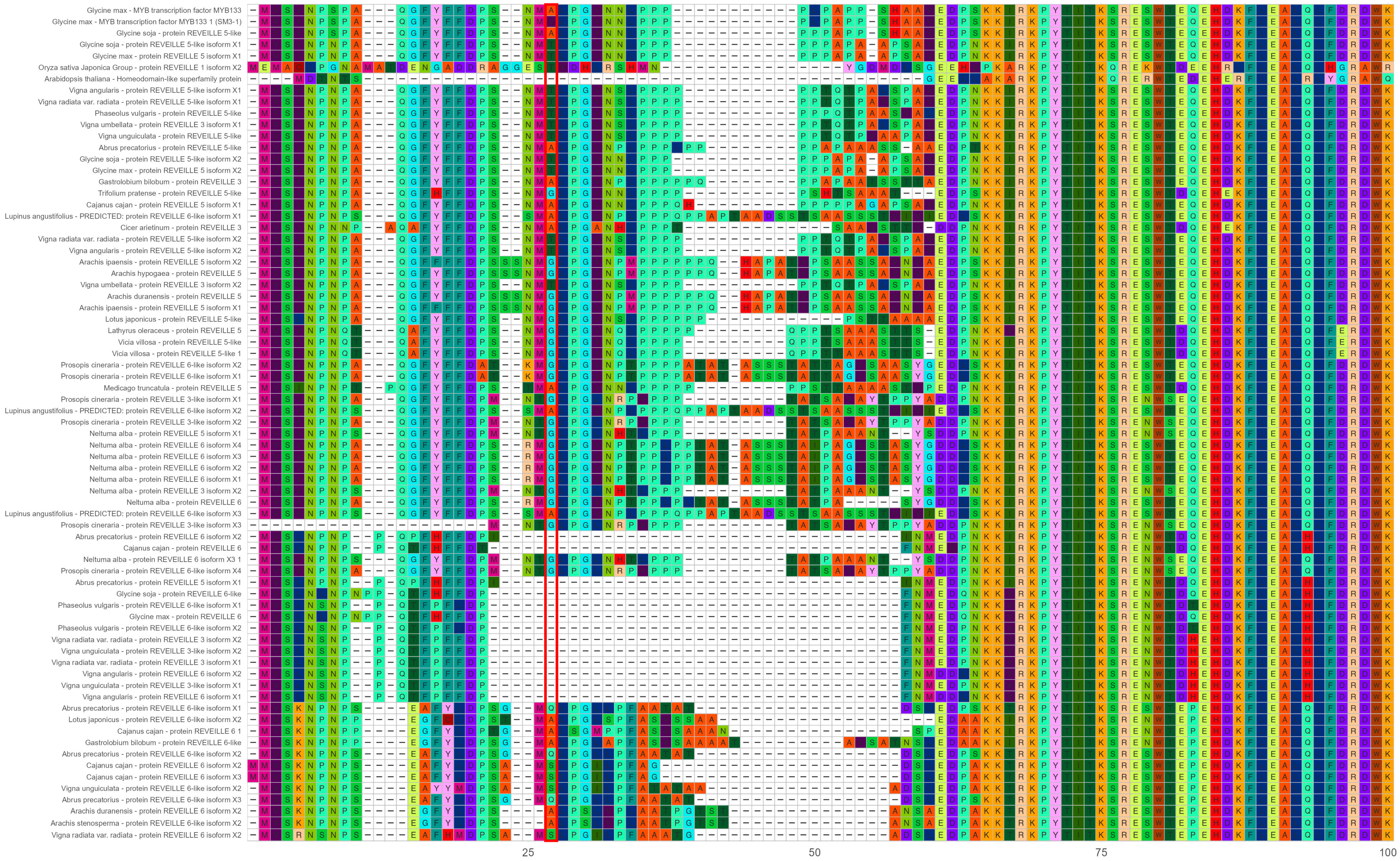

Figure S6.1. MSA of MYB133 homologs highlighting the SNP site at position 21. The alignment comprises 73 homolog sequences.

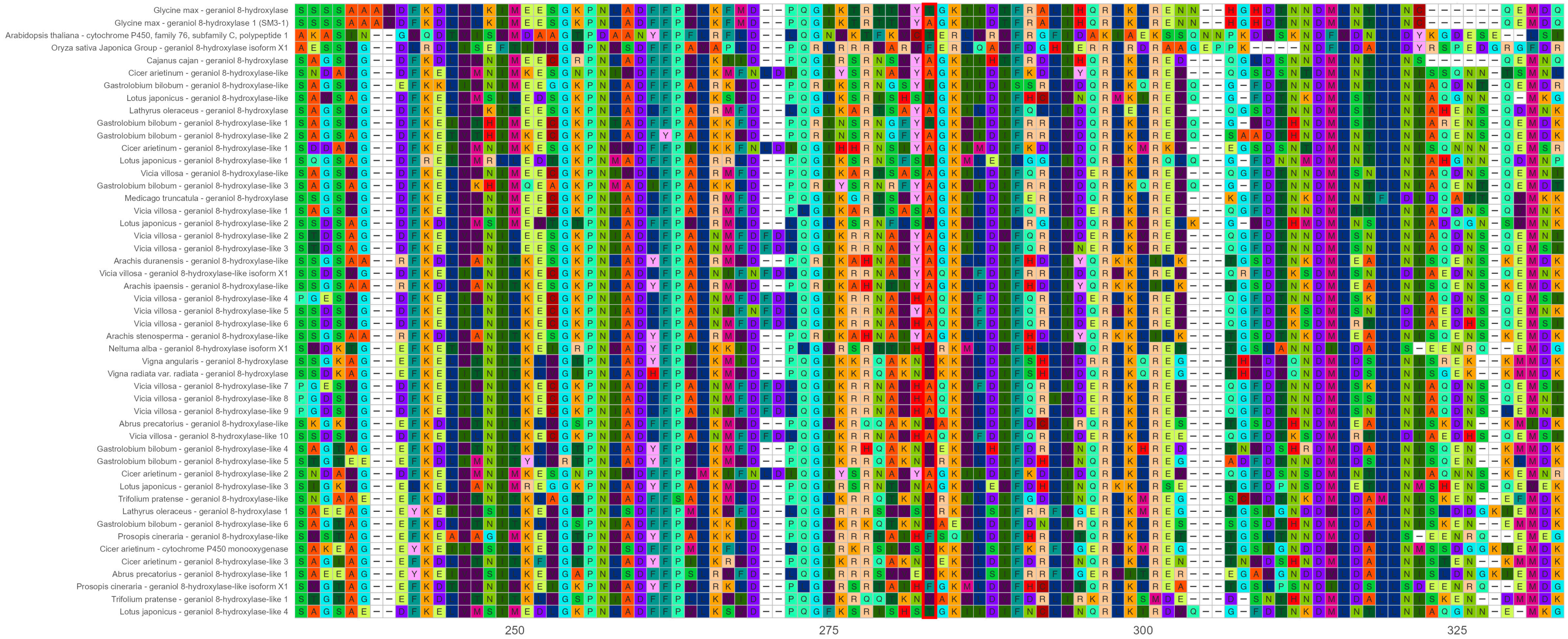

Figure S6.2. MSA of F6H homologs highlighting the SNP site at position 248. The alignment comprises 49 homolog sequences.

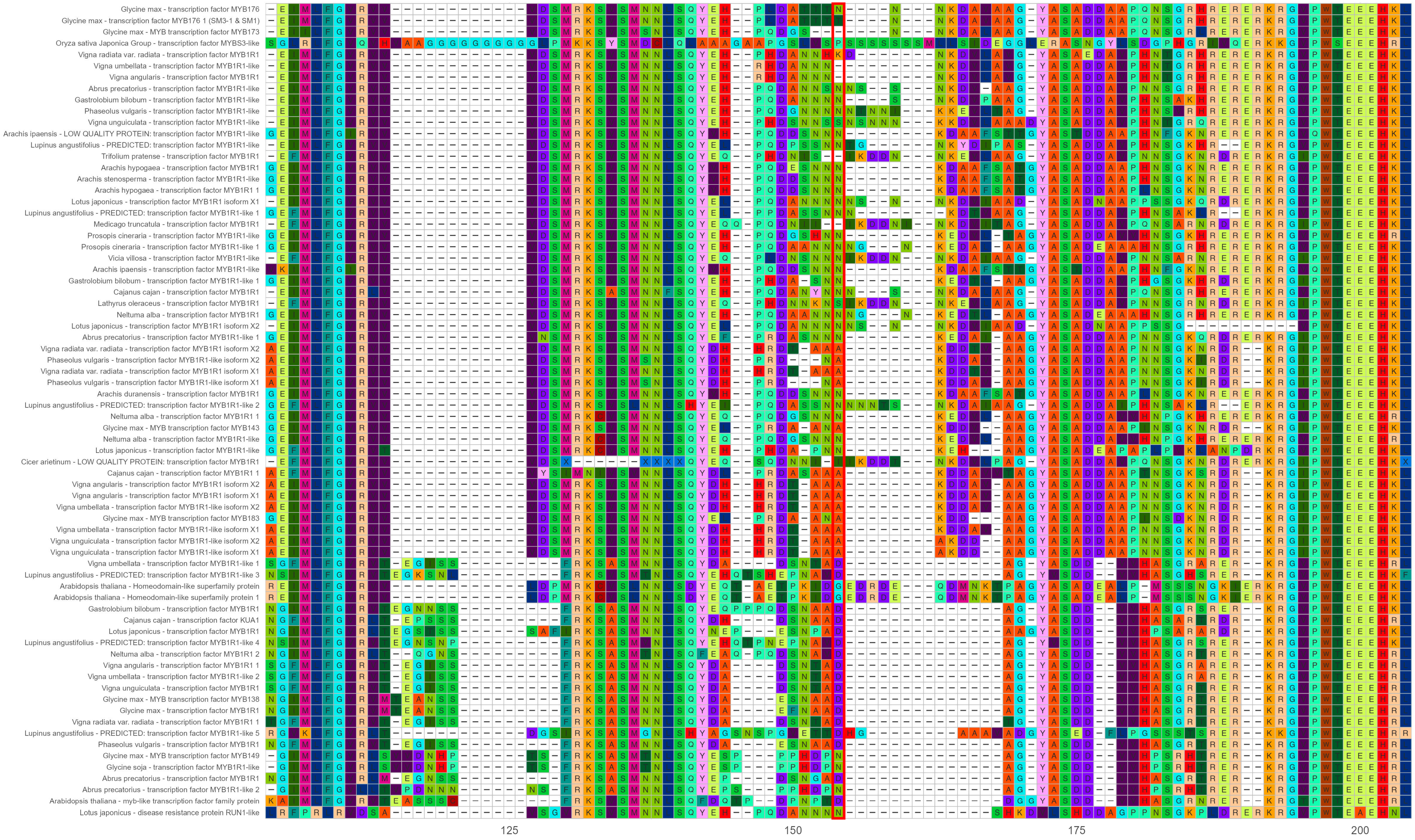

Figure S6.3. MSA of MYB176 homologs highlighting the SNP site at position 46. The alignment comprises 72 homolog sequences.

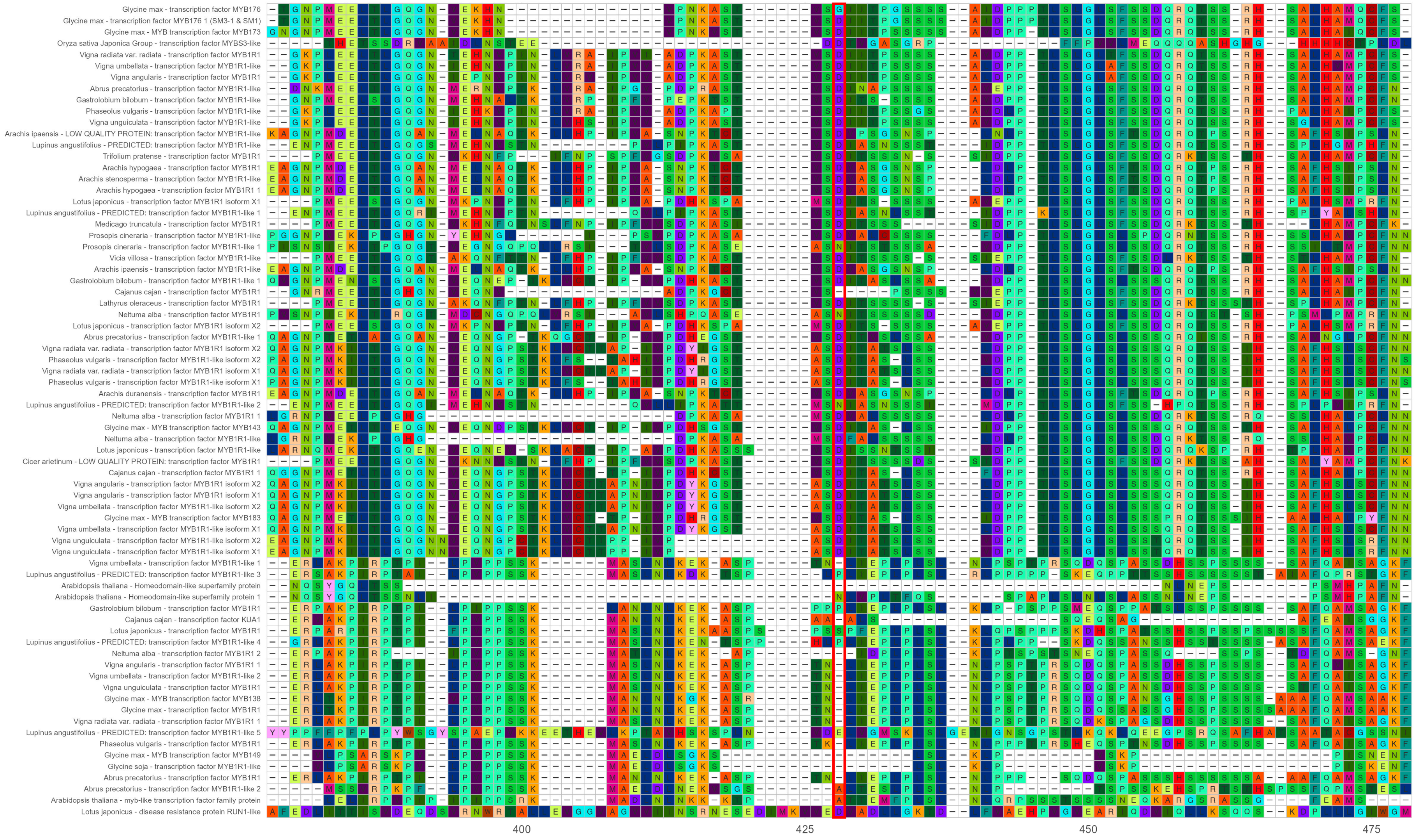

Figure S6.4. MSA of MYB176 homologs highlighting the SNP site at position 232. The alignment comprises 72 homolog sequences.

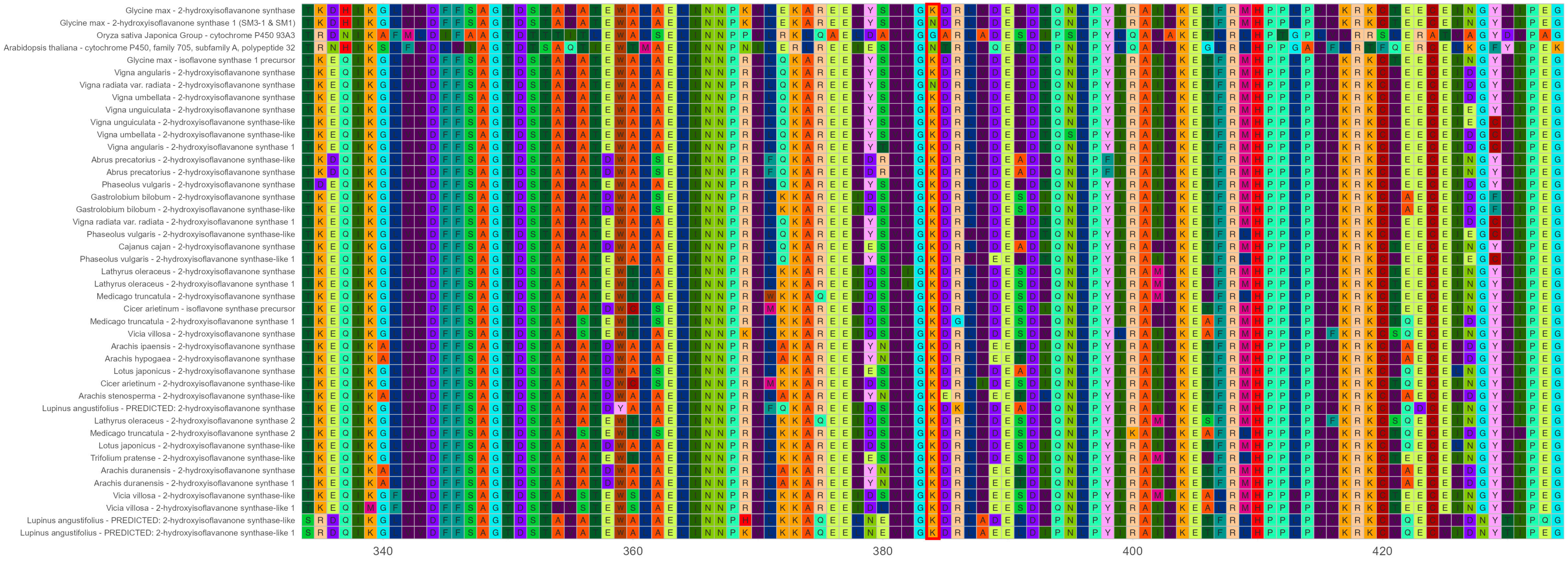

Figure S6.5. MSA of IFS2 homologs highlighting the SNP site at position 340. The alignment comprises 43 homolog sequences.

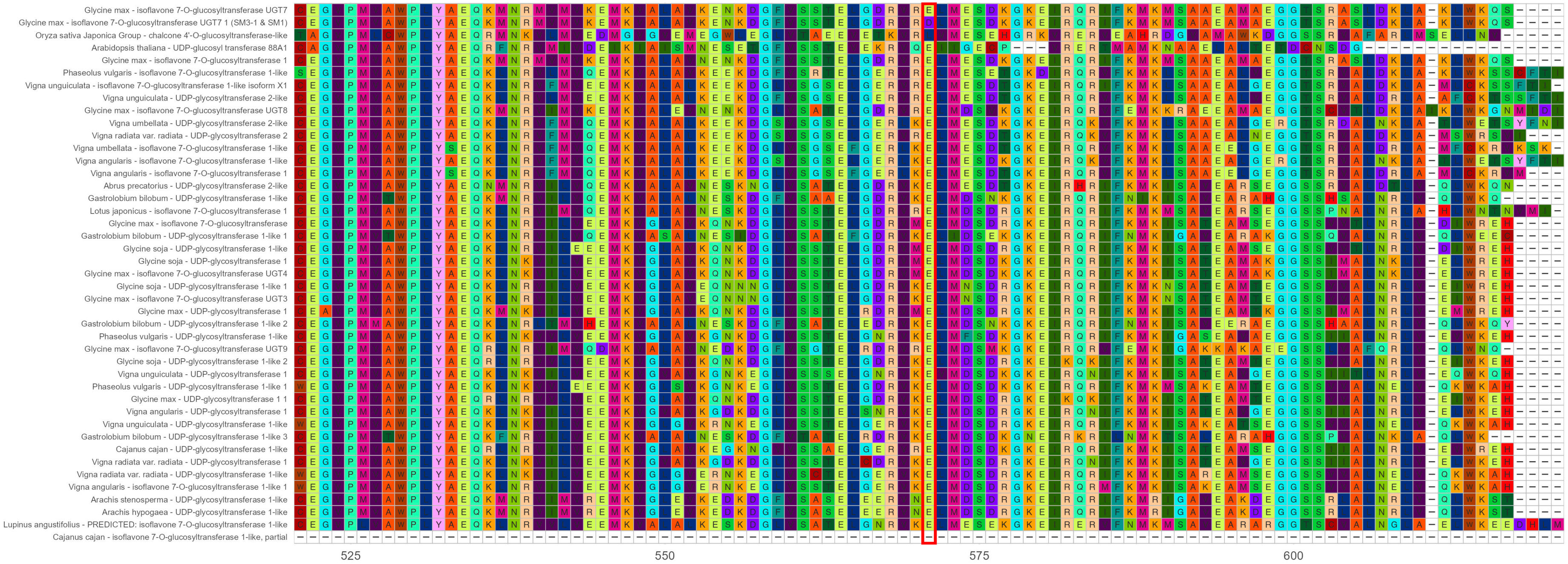

Figure S6.6. MSA of UGT7 homologs highlighting the SNP site at position 428. The alignment comprises 43 homolog sequences.

Homology Modelling

Protein	SWISS-MODEL Template ID	Identity (%)	Coverage	Sequence Similarity	GMQE
MYB133 (reference)	I1MKS3.1.A	90.94	1.00	0.59	0.65
MYB133 (mutant)	I1MKS3.1.A	90.94	1.00	0.59	0.65
F6H (reference)	I1LJ26.1.A	100.00	1.00	0.61	0.92
F6H (mutant)	I1LJ26.1.A	99.61	1.00	0.61	0.92
MYB176 (reference)	D8L1Z5.1.A	100.00	1.00	0.61	0.62
MYB176 (mutant)	D8L1Z5.1.A	99.30	1.00	0.61	0.62
IFS2 (reference)	Q9M6D6.1.A	97.31	1.00	0.60	0.90
IFS2 (mutant)	Q9M6D6.1.A	97.12	1.00	0.60	0.90
UGT7 (reference)	I1MPF3.1.A	100	1.00	0.61	0.92
UGT7 (mutant)	I1MPF3.1.A	99.79	1.00	0.61	0.92

Table S6.1. SWISS-MODEL template selection and quality metrics for reference and mutant homology models of MYB133, F6H, MYB176, UGT7 and IFS2. For each model, the selected template and associated identity, coverage, sequence identity, and GMQE values are provided.
