# Supplementary figures and images for: Comparative Transcriptomic Analysis Reveals Divergent Stress Adaptation Strategies in Gamma-Induced Soybean Mutants
Source: Plants (Basel). 2026 Apr 17;15(8):1241. doi: 10.3390/plants15081241 (PMC13120069; doi:10.3390/plants15081241)

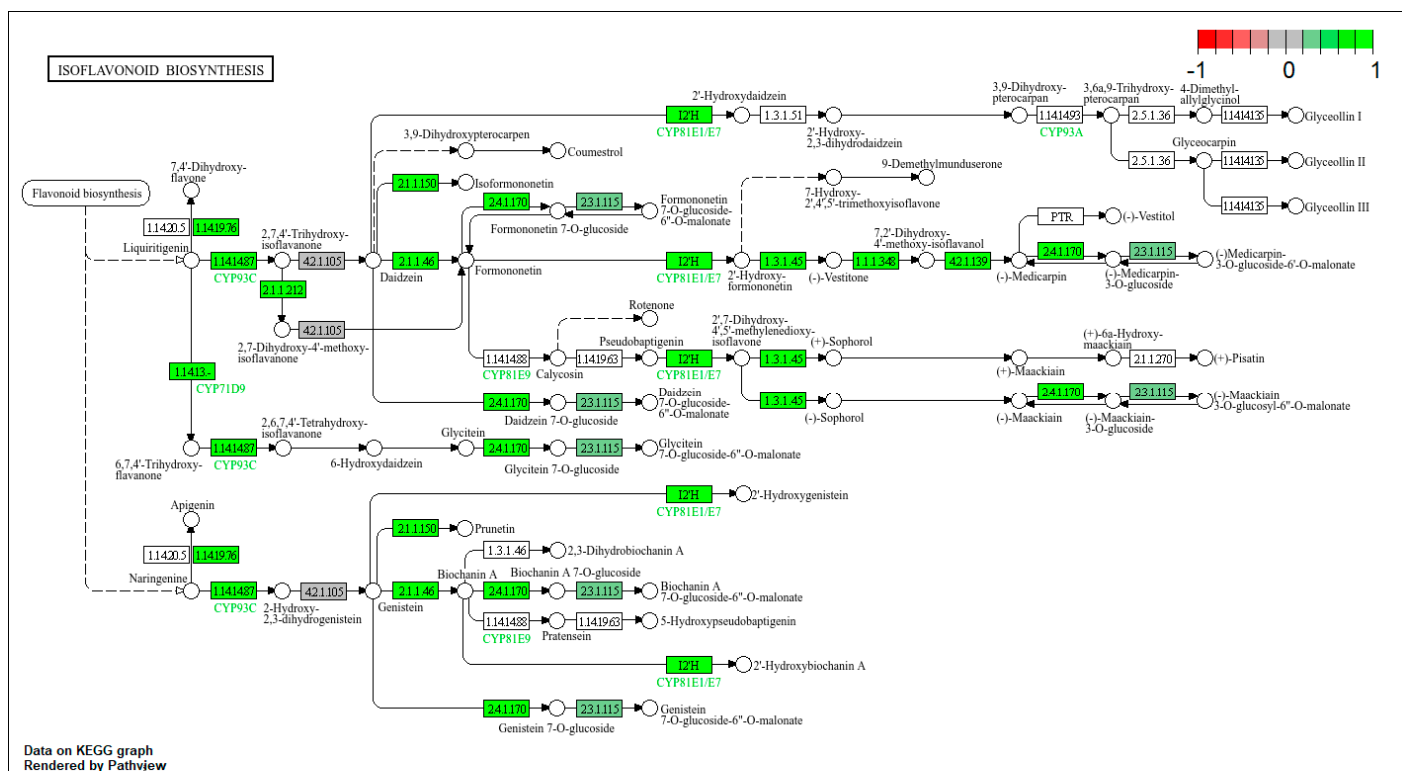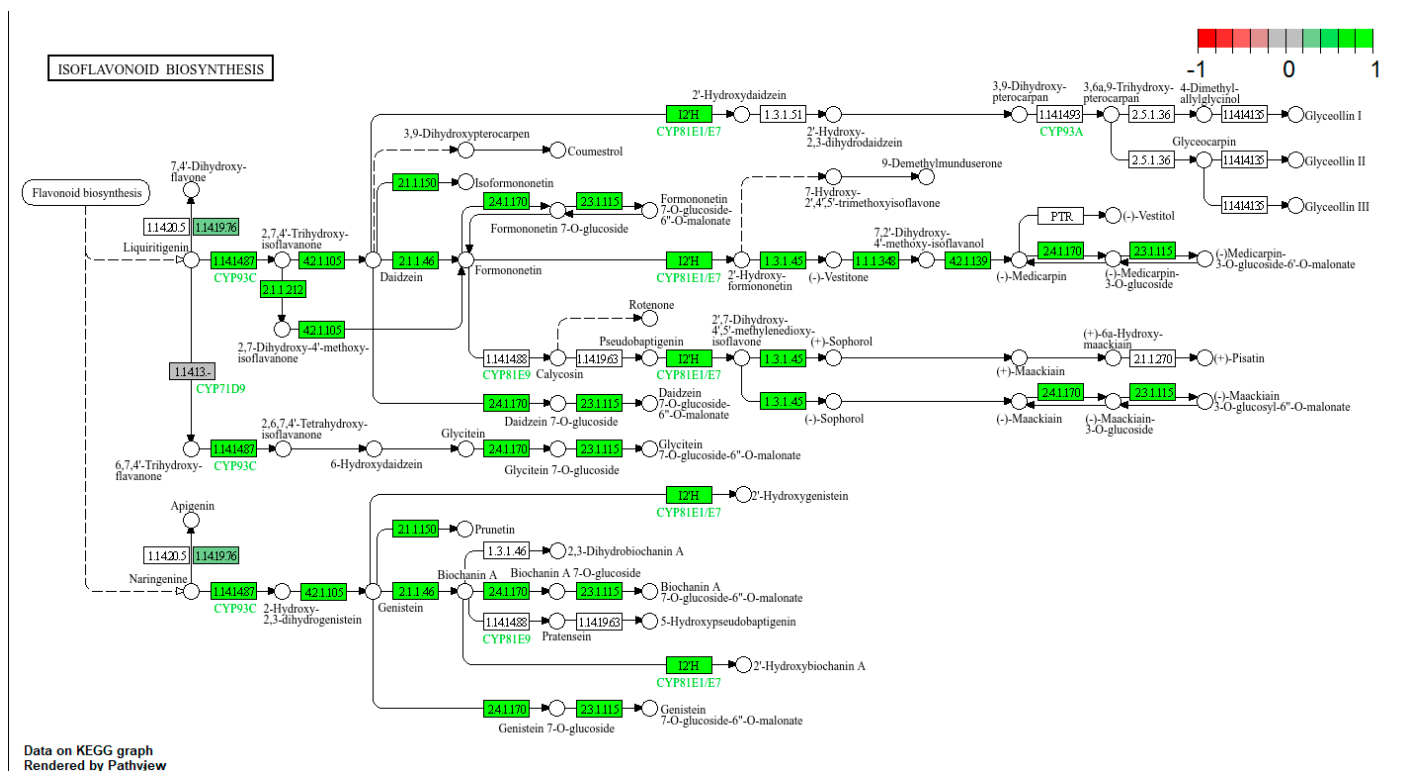

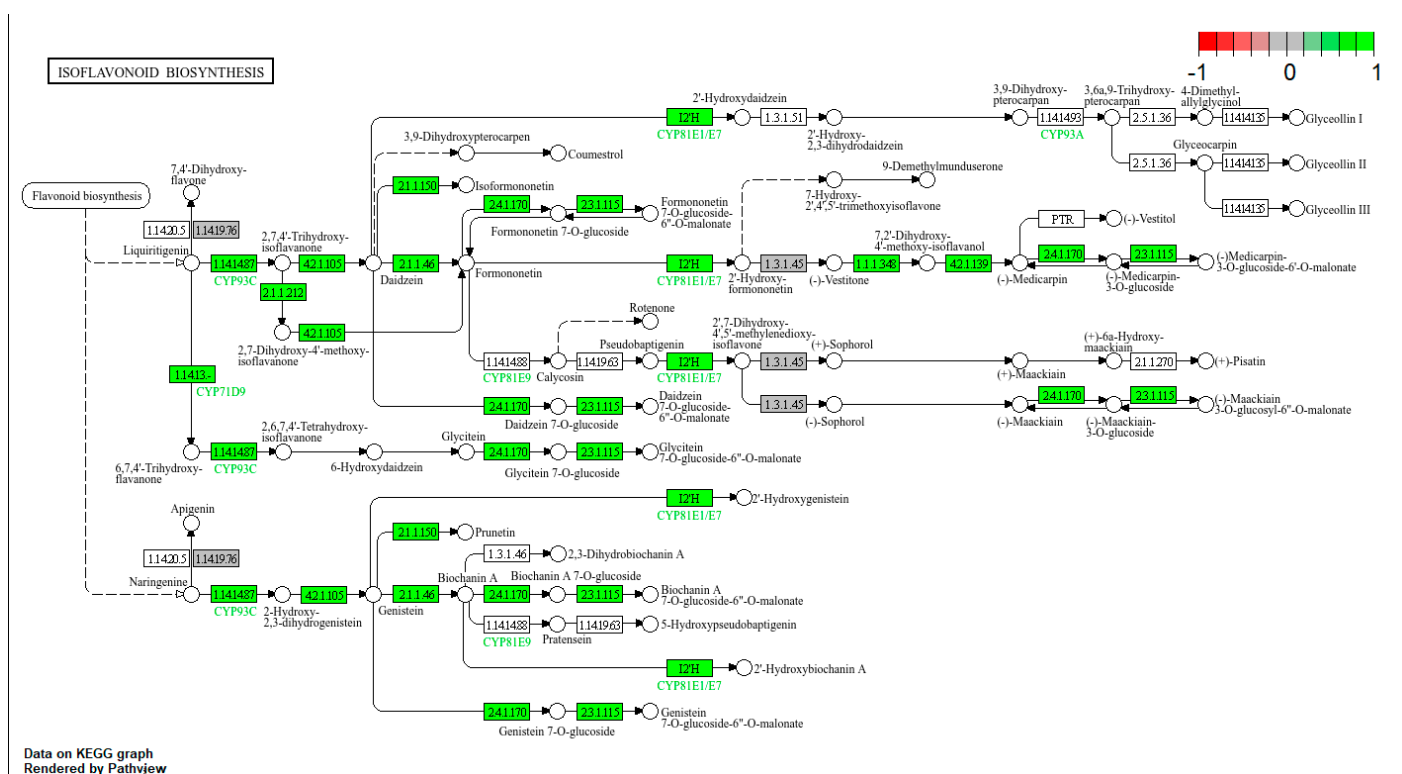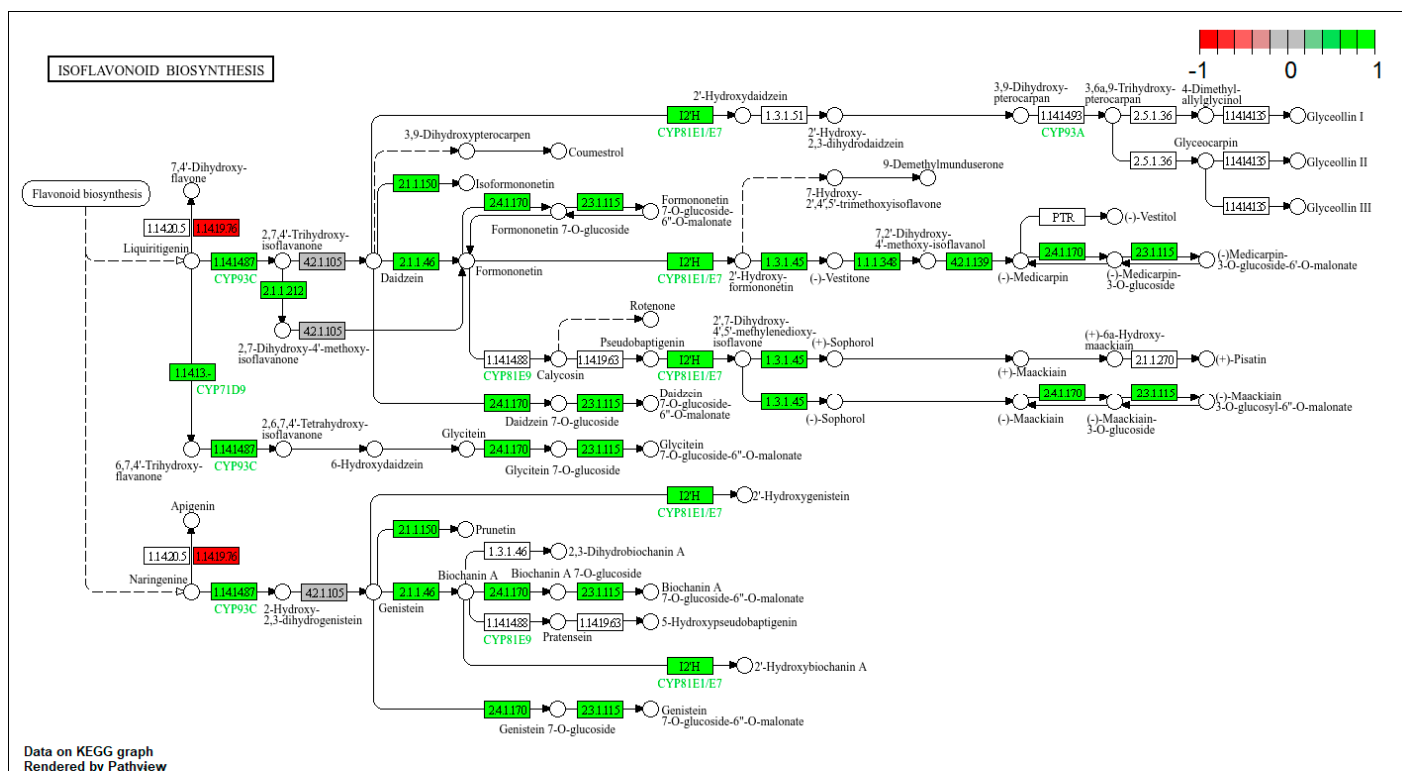

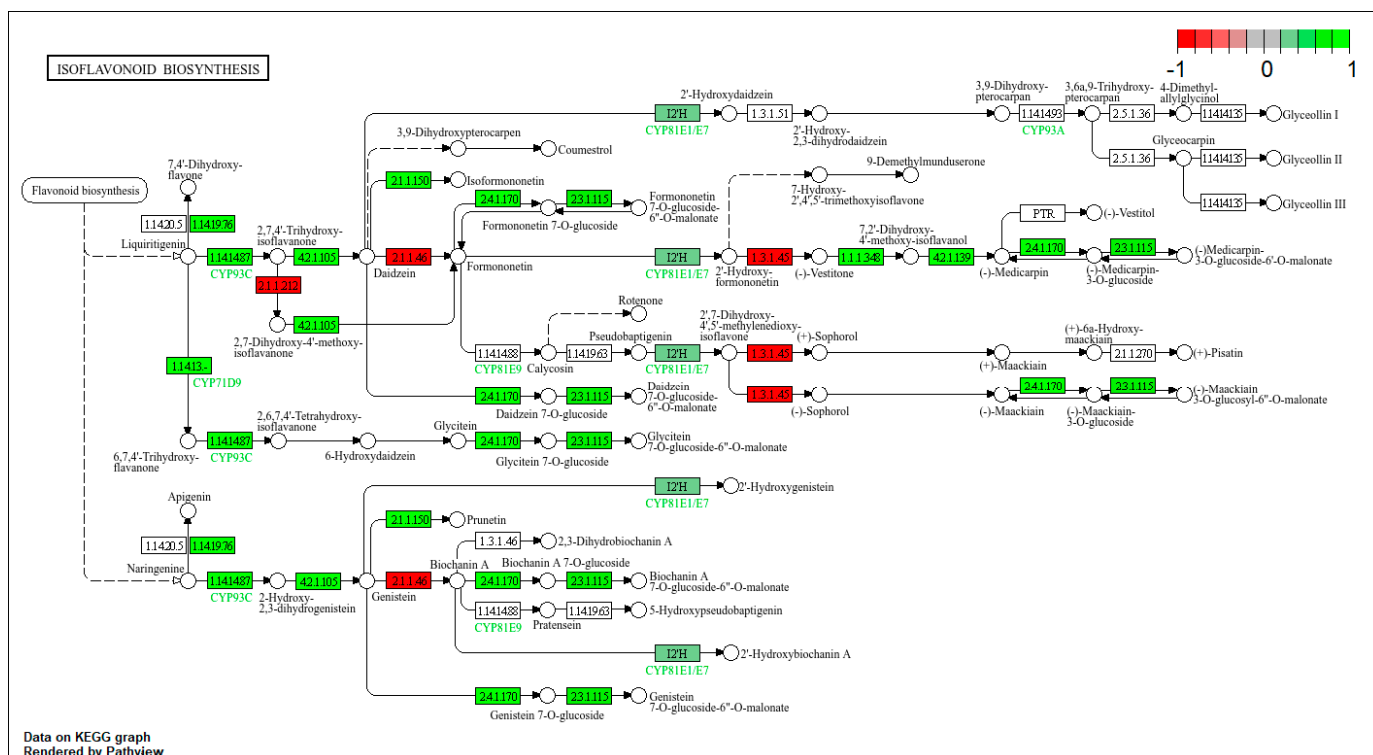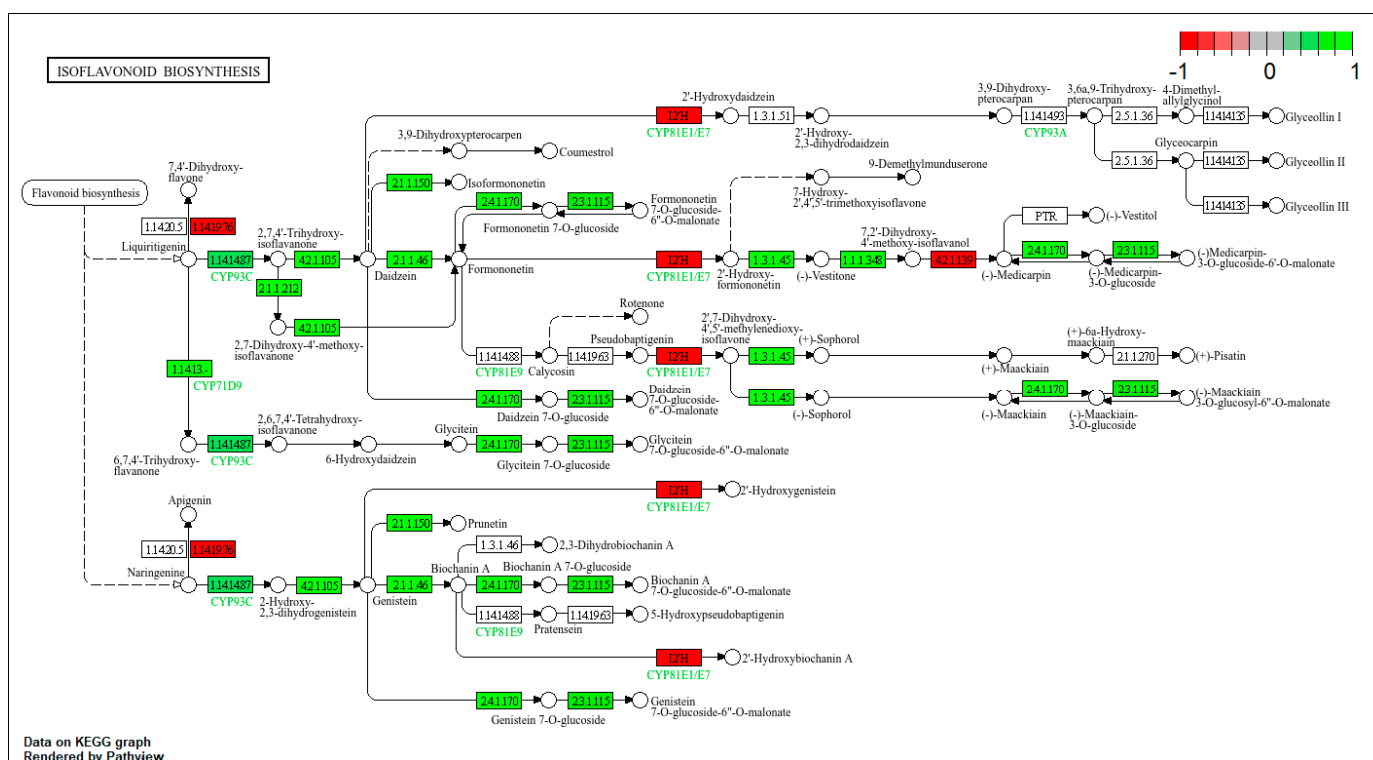

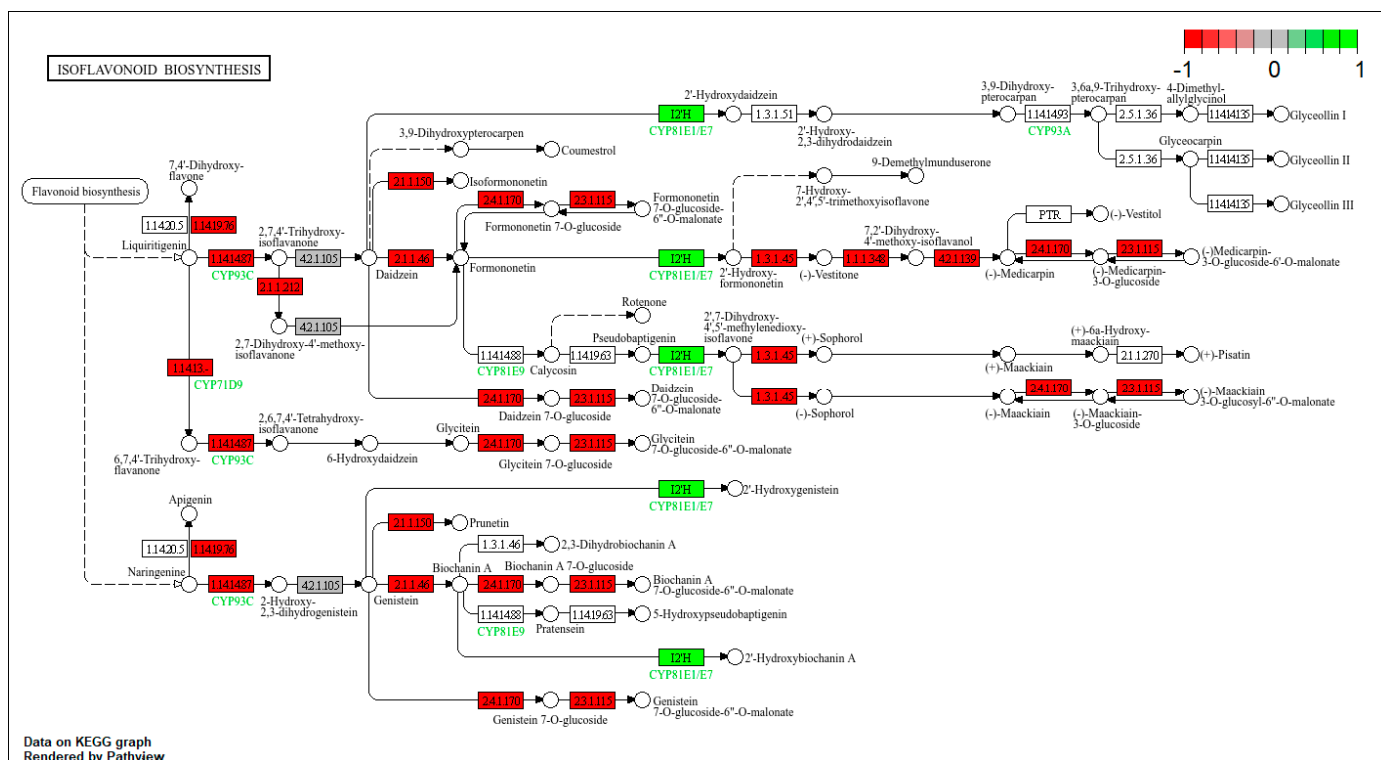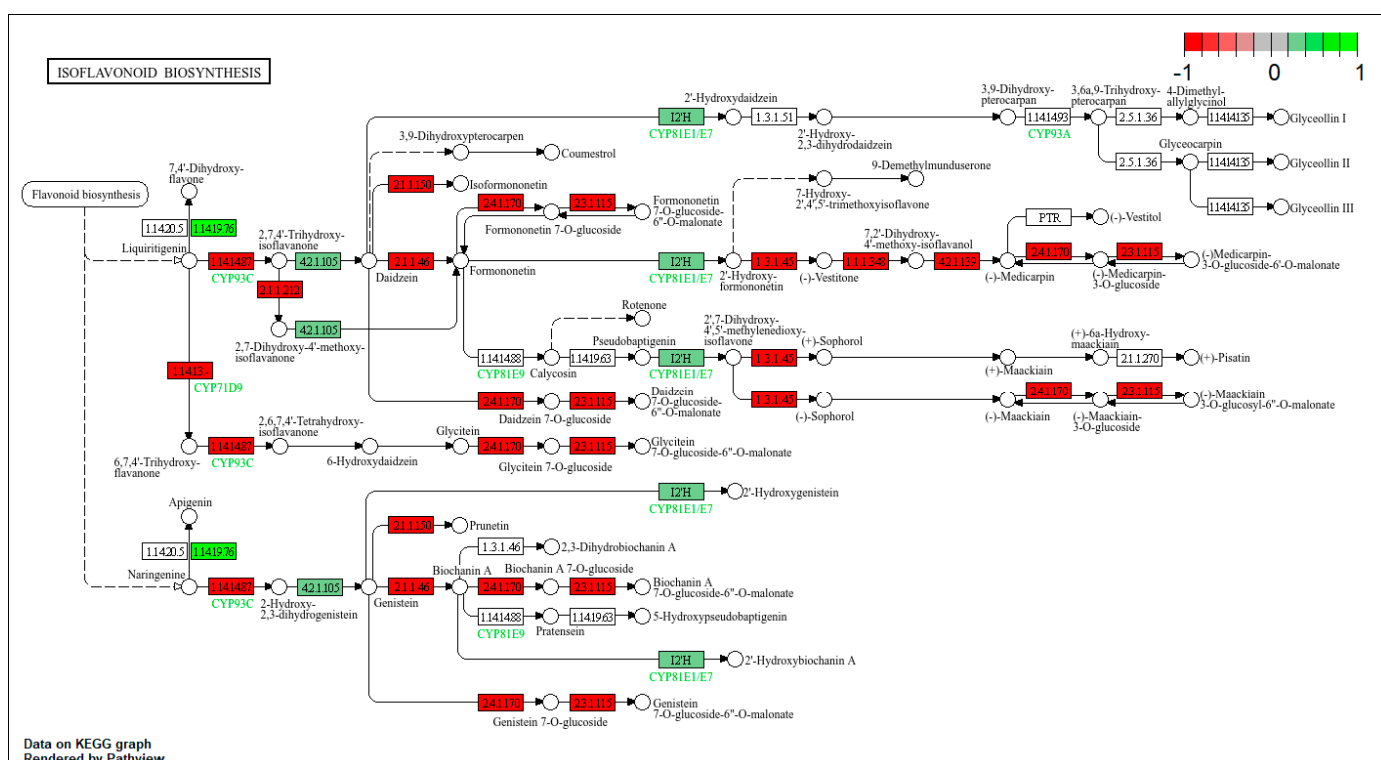

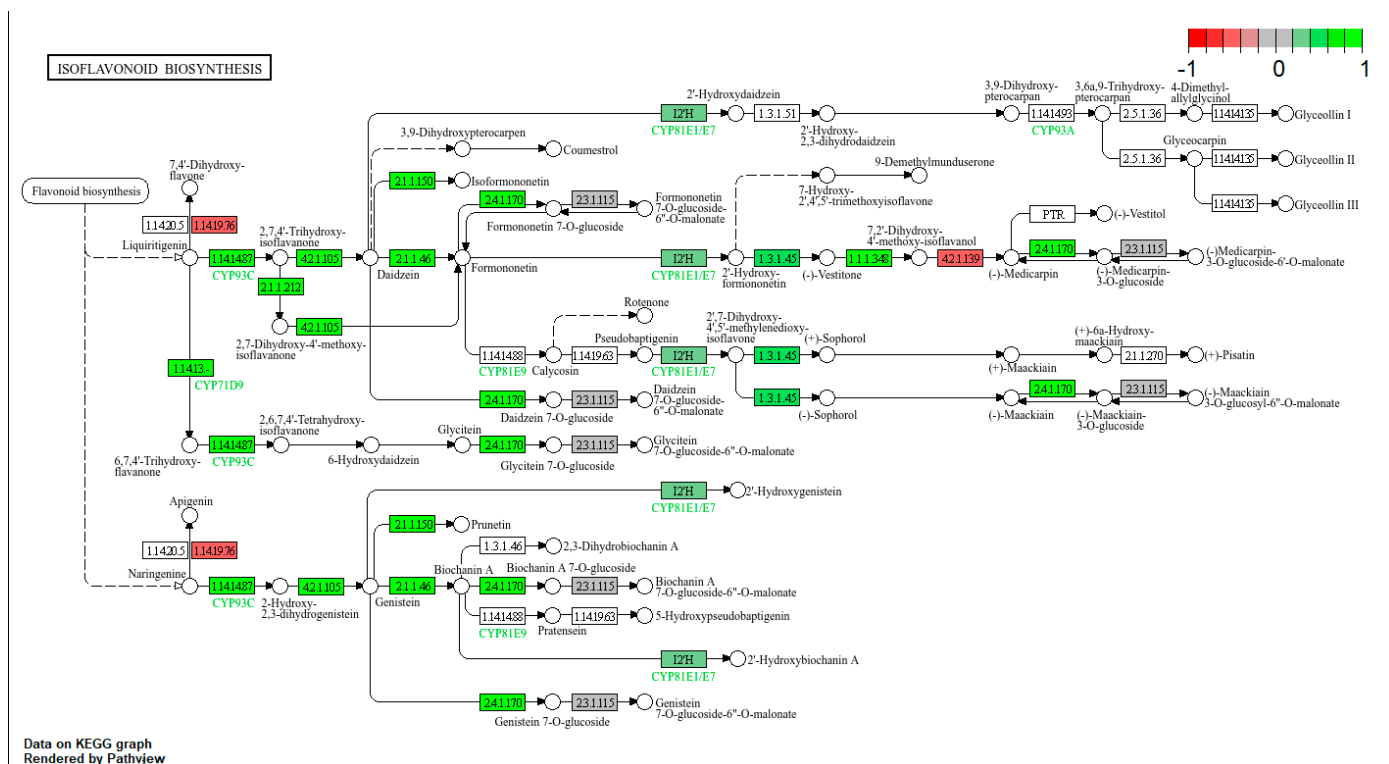

Figure 7C. SM3-1 NaCl vs. untreated SM3-1

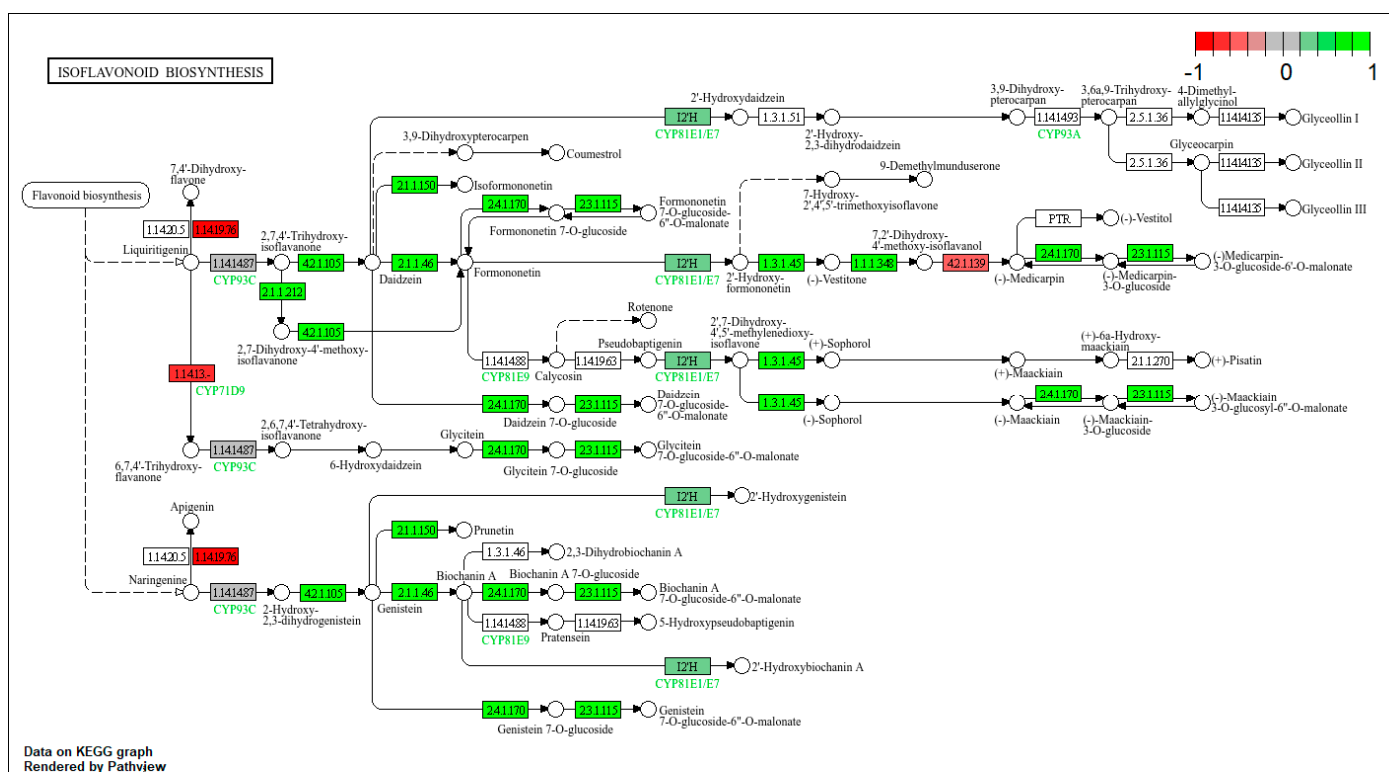

Figure 7D. SM1 NaCl vs. untreated SM1

Supplement: Supplementary file 1 [file plants-15-01241-s001.zip › Supplementary S3.pdf]
